# Evidence for a role of protein phosphorylation in the maintenance of the cnidarian–algal symbiosis

**DOI:** 10.1111/mec.15298

**Published:** 2019-12-06

**Authors:** Fabia Simona, Huoming Zhang, Christian R. Voolstra

**Affiliations:** ^1^ Red Sea Research Center Division of Biological and Environmental Science and Engineering (BESE) King Abdullah University of Science and Technology (KAUST) Thuwal Saudi Arabia; ^2^ Core Labs King Abdullah University of Science and Technology (KAUST) Thuwal Saudi Arabia; ^3^ Department of Biology University of Konstanz Konstanz Germany

**Keywords:** cnidarian–algal symbiosis, data‐independent acquisition, mass spectrometry, phosphorylation‐mediated protein signalling, quantitative phosphoproteomics

## Abstract

The endosymbiotic relationship between cnidarians and photosynthetic dinoflagellate algae provides the foundation of coral reef ecosystems. This essential interaction is globally threatened by anthropogenic disturbance. As such, it is important to understand the molecular mechanisms underpinning the cnidarian–algal association. Here we investigated phosphorylation‐mediated protein signalling as a mechanism of regulation of the cnidarian–algal interaction, and we report on the generation of the first phosphoproteome for the coral model system Aiptasia. Mass spectrometry‐based phosphoproteomics using data‐independent acquisition allowed consistent quantification of over 3,000 phosphopeptides totalling more than 1,600 phosphoproteins across aposymbiotic (symbiont‐free) and symbiotic anemones. Comparison of the symbiotic states showed distinct phosphoproteomic profiles attributable to the differential phosphorylation of 539 proteins that cover a broad range of functions, from receptors to structural and signal transduction proteins. A subsequent pathway enrichment analysis identified the processes of “protein digestion and absorption,” “carbohydrate metabolism,” and “protein folding, sorting and degradation,” and highlighted differential phosphorylation of the “phospholipase D signalling pathway” and “protein processing in the endoplasmic reticulum.” Targeted phosphorylation of the phospholipase D signalling pathway suggests control of glutamate vesicle trafficking across symbiotic compartments, and phosphorylation of the endoplasmic reticulum machinery suggests recycling of symbiosome‐associated proteins. Our study shows for the first time that changes in the phosphorylation status of proteins between aposymbiotic and symbiotic Aiptasia anemones may play a role in the regulation of the cnidarian–algal symbiosis. This is the first phosphoproteomic study of a cnidarian–algal symbiotic association as well as the first application of quantification by data‐independent acquisition in the coral field.

## 1 INTRODUCTION

Scleractinian corals are reef‐building architects that provide the foundation of one of the most diverse marine ecosystems, coral reefs (Reaka‐Kudla, Wilson, & Wilson, 1997). Corals are cnidarian animals that rely upon a functional symbiosis with their intracellular, photosynthetic dinoflagellate algae in the family Symbiodiniaceae (Davy, Allemand, & Weis, 2012; LaJeunesse et al., 2018). The endosymbiotic algae supply the coral with metabolites by translocating substantial amounts of photosynthetically fixed carbon to the host (Davy, Lucas, & Turner, 1996; Kopp et al., 2015; Muscatine, Falkowski, Porter, & Dubinsky, 1984). In return, the coral provides lipids, amino acids and substrates for photosynthesis (Imbs, 2013; Wang & Douglas, 1998), as well as a light‐rich and sheltered environment to the microalgae (Enríquez, Méndez, & Prieto, 2005; Falkowski, Dubinsky, Muscatine, & Porter, 1984). This symbiotic interaction is essential for the coral holobiont, which encompasses a broad suite of coral‐associated microbes (e.g., bacteria, archaea, viruses and fungi), allowing it to thrive and flourish in oligotrophic waters as well as to support highly biodiverse ecosystems (Jaspers et al., 2019; Rohwer, Seguritan, Azam, & Knowlton, 2002).

Coral reefs are globally under threat due to environmental and anthropogenic pressure. The causes of coral decline, among others, are increasing sea surface temperatures, ocean acidification and water eutrophication (Hoegh‐Guldberg et al., 2007; Rädecker, Pogoreutz, Voolstra, Wiedenmann, & Wild, 2015), which can lead to the breakdown of the coral–algal association and subsequent loss of the microalgae, a process known as coral bleaching. It is therefore crucial to better understand the molecular mechanisms and regulation of the coral–algal association. Notably, studying the cnidarian–dinoflagellate symbiosis in corals is difficult for several reasons, including the challenges with maintaining corals in aquarium settings, long reproductive cycles and the obligate dependency on their symbiotic association, that is, the absence of a “control” aposymbiotic (symbiont‐free) state (Voolstra, 2013). To this end, the sea anemone Aiptasia (sensu* Exaiptasia pallida*) is becoming increasingly popular as a model system for the study of coral–algal symbiosis (Baumgarten et al., 2015; Lehnert, Burriesci, & Pringle, 2012; Rädecker et al., 2018; Wolfowicz et al., 2016). Aiptasia is easily reared in laboratory tanks, has a short asexual reproductive cycle and can be kept in an aposymbiotic state, enabling direct comparison between symbiotic and aposymbiotic conditions. In corals, this type of comparison is only possible in larvae, which are commonly aposymbiotic and acquire their symbionts horizontally (Dubinsky & Stambler, 2010; Schnitzler & Weis, 2010; Voolstra et al., 2009).

Comparison of aposymbiotic and fully symbiotic Aiptasia at the transcriptomic, proteomic and metabolic level revealed regulation of host biological processes that include metabolism, nutrient transport, energy storage and vesicle trafficking (Baumgarten et al., 2015; Ganot et al., 2011; Lehnert et al., 2014; Matthews et al., 2017; Oakley et al., 2016; Sproles et al., 2019). The marked overlap in the patterns at different molecular levels suggests that these biological processes are likely to be critical in defining the symbiotic phenotype. Because proteins are the functional unit of the cell and more directly relate to the prevalent phenotype than expressed genes, we investigated the role of protein signalling in the cnidarian–algal interaction. We focused on protein phosphorylation, a post‐translational modification (PTM) with a prevalent role in the control of protein activity and signal transduction (Aivaliotis et al., 2009; Humphrey, James, & Mann, 2015).

In eukaryotes, at least one‐third of the total proteome is phosphorylated mainly on hydroxyl amino acids (serine, threonine, tyrosine), and highly conserved cellular processes are thereby regulated (Jünger & Aebersold, 2014; Ochoa et al., 2016). Among others, protein phosphorylation is commonly employed to accommodate reversible adjustments in metabolism, sugar transport, oxidative stress responses, control of heat‐shock and adaptation to light (Cozzone, 2005). Phosphorylation has also been described in pathogenic symbioses, where changes in the host cellular structure and function occur as a consequence of intracellular pathogens surviving within the invaded cell (Cozzone, 2005; Schmutz et al., 2013). In this regard, more recent work in corals alludes to “selfish algal symbionts” (Aranda et al., 2016; Nielsen, Petrou, & Gates, 2018; Rädecker et al., 2018; Sproles et al., 2019). Thus, protein phosphorylation is an attractive target to explore in order to better understand the mechanistic underpinnings of the cnidarian–algal interaction.

In this study, we used mass spectrometry (MS)‐based phosphoproteomics to unravel the phosphorylation dynamics in the cnidarian–algal association. To do this, we first generated a reference phosphoproteome spectral library using data‐dependent acquisition (DDA or shotgun proteomics), which provided comprehensive phosphoproteome coverage across aposymbiotic and symbiotic conditions in Aiptasia polyps. The spectral library served to retrieve the phosphopeptide identity during quantification using DIA (data‐independent acquisition)‐based SWATH‐MS (sequential windowed acquisition of all theoretical fragment ion mass spectra), known to enable highly accurate and reproducible quantification of protein phosphorylation. To allow for discrimination between translational regulation (de novo protein synthesis) and PTM (addition/removal of phosphate groups to existing proteins), we additionally generated a total proteome data set used for normalization of phosphorylation against protein amount. In doing so, we found characteristic phosphoproteomic profiles distinguishing aposymbiotic and symbiotic states and differential phosphorylation targeting biological processes that have not been previously described in the context of the cnidarian–algal symbiosis, namely “phospholipase D signalling pathway” and “protein processing in the endoplasmic reticulum.” We suggest that changes in the phosphorylation status of these signalling pathways have a putative role in the control of an established cnidarian–algal association. More broadly, the finding of extensive regulation at the level of the phosphoproteome may argue for dynamic control of the symbiotic state.

## 2 MATERIALS AND METHODS

### 2.1 Aiptasia rearing

Aposymbiotic and symbiotic Aiptasia anemones of the clonal strain CC7 (Baumgarten et al., 2015) were reared in incubators at 25°C, in 0.5–1‐L tanks filled with freshly collected, autoclaved, 0.22‐μm‐filtered (Cat. GSWP04700; MF‐Millipore) seawater (AFSW) from the Red Sea with salinity adjusted to 37 PSU, and fed *Artemia* twice weekly. Aposymbiotic Aiptasia were generated by repetitive cold‐shock of 4‐hr cycles in AFSW at 4°C and treatment with 50 μm of the photosynthetic inhibitor diuron (Cat. D2425; Sigma‐Aldrich). Aposymbiotic anemones were kept in a dark incubator for more than 1 year and regularly examined for residual symbiont recolonization/replication under a fluorescence microscope (Leica DMI3000 B) (Figure S1). Part of the aposymbiotic population was recolonized with the *Breviolum minutum* strain SSB01 (clade B) (LaJeunesse et al., 2018; Xiang, Hambleton, DeNofrio, Pringle, & Grossman, 2013). We additionally assessed symbiont presence/absence in Aiptasia batches by quantitative polymerase chain reaction (qPCR) using ITS2 primers that discriminate between *Symbiodinium* spp. (clade A) and *B. minutum* (clade B). We tested for these two clades because the former is the native symbiont of CC7, while the latter was used for recolonization. In both instances, the qPCR did not produce amplicons with 30 cycles, thus confirming that animals were aposymbiotic (Table S1). Full recolonization occurred in about 30 days at 12 hr of light exposure. To avoid *Artemia* contaminations, the food supply was ceased 5 days before Aiptasia sampling.

### 2.2 Tissue lysis, protein extraction and digestion

**Figure 1:**
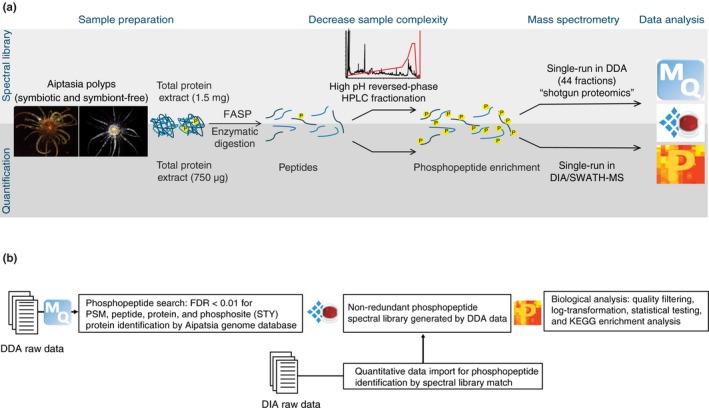
(a) Aiptasia sample preparation workflow for phosphoproteomics. Proteins were extracted from aposymbiotic and symbiotic Aiptasia polyps and digested in‐column. For spectral library generation, peptides were HPLC‐fractionated before phosphopeptide enrichment, and MS acquisition was performed in DDA mode. For phosphopeptide quantification no HPLC fractionation was performed and acquisition was performed in DIA/SWATH‐MS. (b) Data processing and analysis workflow. The DDA raw files were imported into MaxQuant for phosphopeptide search and used for spectral library generation in Spectronaut X. The quantitative DIA data were imported into Spectronaut X for retrieval of phosphopeptide identity by spectral library match. Biological and statistical data analysis was conducted in Perseus

The detailed protocol describing sample preparation and data acquisition for both phosphopeptide spectral library generation and phosphopeptide quantification in Aiptasia is publicly available (Simona, Zhang, & Voolstra, 2018). Briefly, for the spectral library, a pool of 20 small‐sized (2‐mm basal disc) anemones per experimental condition (aposymbiotic and symbiotic) were collected and separately processed throughout the entire protocol. For quantification, each biological replicate per condition (five aposymbiotic, five symbiotic) was constituted by a pool of 10 small‐sized anemones. Samples were lysed in 8 M urea buffer supplemented with protease (Cat. 4693159001; Roche Applied Science) and phosphatase (Cat. 04906845001; Roche Applied Science) inhibitors, using a Tenbroeck Tissue Grinder (Cat. 357421; Wheaton) and an Ultrasonic Processor (Thomas Scientific). Proteins were extracted by methanol–chloroform precipitation and measured via a Pierce Micro BCA Protein Assay (Cat. 23235; Thermo Fisher Scientific) according to the manufacturer's instructions. For the spectral library, 1.5 mg of total protein extract per experimental condition was used, while for quantification 750 µg of total protein extract per biological replicate was sufficient. Protein digestion was performed by filter‐aided sample preparation (FASP), which improved the purity of the eluted peptides (Wiśniewski, Zougman, Nagaraj, & Mann, 2009). Briefly, the protein extract was loaded on a 10‐kDa filter unit (Cat. 42407; Millipore), reduced in 10 mM dithiothreitol (DTT), alkylated in 50 mM iodoacetamide (IAA), and digested with a Trypsin/Lys‐C mix (Cat. V5071; Promega Corp.) at a 1:50 enzyme/protein ratio (w/w), at 37°C overnight. The peptide elute was acidified for desalting in a reversed‐phase C18 Sep‐Pak cartridge (Cat. WAT023590; Waters Corp.), containing an oligo R3 reversed‐phase resin (Cat. 1133903; Applied Biosystems). For spectral library generation, peptide elution from the Sep‐Pak cartridge was performed in 75% acetonitrile (ACN)/0.1% trifluoroacetic acid (TFA), a solution compatible with the following chromatographic fractionation. For quantification by DIA/SWATH‐MS, peptides were eluted in a solution containing 80% ACN, 5% TFA and 1 M of freshly added glycolic acid that allowed direct phosphopeptide enrichment (Figure 1a).

### 2.3 Peptide fractionation

Deep coverage of the phosphopeptide spectral library was obtained by high pH reversed‐phase fractionation according to the protocol of Batth &  Olsen (2016). Briefly, an XBridge Peptide BEH C18 column (Cat. 186003570; Waters Corp.) was connected to a Surveyor Plus high‐performance liquid chromatography (HPLC) system (Thermo Fisher Scientific) and a 120‐min gradient of buffer B (90% ACN, 5 mM ammonium hydroxide) in buffer A (5 mM ammonium hydroxide) was designed as follows: from 0%–5% to 25% of buffer B within 60–90 min, from 25% to 40% of buffer B within 5–10 min, ramp up to 60%–70% of buffer B within 5 min, maintain at 70% for 5 min before ramping down to 0%–5% of buffer B. A total of 110 fractions were collected and reconcatenated orthogonally (by mixing different parts of the gradient) into 25 fractions, and their volume was reduced to ~50 µl with a SpeedVac system (Thermo Fisher Scientific).

### 2.4 Phosphopeptide enrichment

TiO_2_ bead‐based phosphopeptide enrichment was performed according to the protocol of Engholm‐Keller et al. (2012). First, desalted and/or fractionated peptides were brought to a volume of 1 ml with 80% ACN, 5% TFA and 1 M of freshly added glycolic acid. Then, peptides and TiO_2_ beads (Cat. 502075000; GL Sciences) were incubated at a 1:6 ratio (w/w), shaking for 10 min in a vortex mixer. After supernatant removal, the TiO_2_‐bound phosphopeptides were washed in 80% ACN/1% TFA followed by 20% ACN/0.5% TFA. After complete evaporation of the solvent, the phosphopeptides were eluted from the dry TiO_2_ beads by incubation in 4% ammonia solution in water, pH 11 (32% ammonia stock solution, Cat. 21192.323; VWR), rocking for 10 min. The phosphopeptide‐rich supernatant was acidified with formic acid (FA) (14% by vol.) and TFA (4% by vol.) and transferred on top of a homemade C18 STAGE tip assembled and preconditioned according to the protocol developed by Rappsilber, Mann, and Ishihama (2007). After two washes with 0.1% TFA in water, phosphopeptides were eluted twice in 75% ACN/0.1% TFA and completely dried via a SpeedVac.

### 2.5 Phosphopeptide preparation for LC‐MS/MS

Prior to liquid chromatography coupled to tandem mass spectrometry (LC‐MS/MS) injection, phosphopeptides were resuspended in 0.1% FA, 0.1% ACN and LC‐MS grade water solution and sonicated in an ultrasonic cleaner. Phosphopeptides were measured at *A*_280_ by NanoDrop (Thermo Fisher Scientific) and concentrations were normalized across samples for quantitative DIA/SWATH‐MS analysis. Indexed retention time (iRT) standards (Cat. Ki‐3002; Biognosys) were added to the ready‐to‐inject phosphopeptide mixture at a 1:10 ratio (v/w) for DDA MS (shotgun proteomics), and at a 3:10 ratio (v/w) for multiplexed analysis in DIA/SWATH‐MS. A total of One μg of the peptides was injected in the LC‐MS/MS.

### 2.6 LC‐MS/MS acquisition in DDA

Prior to DDA MS, phosphopeptides were separated with an UltiMate 3000 RSLCnano UHPLC system (Thermo Fisher Scientific) on a 25‐cm Acclaim PepMap 100 C18 column (Cat. 164261; Thermo Fisher Scientific) at a flow rate of 300 nl/min, and over the following 75‐min gradient of buffer B (0.1% FA in 80% ACN) in buffer A (0.1% FA, 0.1% ACN, in LC‐MS grade water): from 5% to 40% of buffer B within 55 min, ramp up to 90% of buffer B within 5 min and maintain at 90% for 5 min, ramp down to 2% of buffer B and maintain for another 10 min. The nano‐LC (nLC) system was connected to a Q‐Exactive HF mass spectrometer (Thermo Fisher Scientific). The phosphopeptides were injected in the spectrometer with a Nonospray Flex ion source with electrospray potential of 1.9 kV and the temperature of the ion transfer tube set at 275°C. A full MS scan (350–1,400 *m*/*z*) was acquired at a resolution of 60,000 (at 200 *m*/*z*) in profile mode and with a target value of 3 × e^6^. The maximum ion accumulation time was set at 100 ms, and charge state screening for precursor ions was activated. The 10 most intense ions (threshold > 2 × e^4^) carrying multiple charges were selected for fragmentation by higher energy collision dissociation (HCD). The dd‐MS2 resolution was set at 15,000 and dynamic exclusion for HCD fragmentation at 20 s. Other settings for fragment ions included a maximum ion accumulation time of 100 ms, target value of 1 × e^5^, normalized collision energy at 28%, and isolation width of 1.8.

### 2.7 LC‐MS/MS acquisition in DIA/SWATH‐MS

Prior to DIA/SWATH‐MS, phosphopeptides were separated on a similar nLC system as for DDA, but connected to a 50‐cm EASY‐Spray column PepMap RSLC C18 (Cat. ES803; Thermo Fisher Scientific) at a flow rate of 300 nl/min, and over the same 75‐min gradient used in DDA. The nLC system was coupled to a Fusion Lumos Orbitrap mass spectrometer (Thermo Fisher Scientific). The sample was introduced into the spectrometer through an EASY‐Spray ion source with an electrospray potential of 1.9 kV and ion transfer tube temperature at 275°C. A full MS scan (400–1,200 *m*/*z* range) was acquired at a resolution of 60,000 in profile mode, with maximum injection time of 20 ms and target value of 5 × e^5^. The instrument was set in DIA mode with optimized quadrupole settings for 32 precursor ion selection windows (each 25 Da wide) over the precursor mass range. Fragmentation by HCD was set at 30%, dd‐MS2 resolution at 30,000, maximum injection time of 50 ms and a target value of 2 × e^5^.

### 2.8 Phosphopeptide spectral library generation from DDA data

DDA raw files were processed with MaxQuant (version 1.6.2.10) using the integrated Andromeda engine for phosphopeptide search (Figure 1b). A false discovery rate (FDR) cutoff of 0.01 was applied at the peptide, protein and modification level. The search included carbamidomethylation (C) as a fixed modification, and phosphorylation (STY), oxidation (M) and acetylation (N‐term) as variable modifications. Peptides with a minimum length of seven amino acids and a maximum of five modifications were considered. Trypsin/P was selected as the digestion enzyme, allowing a maximum of two missed cleavages. Phosphopeptide identity retrieval was done by sequence alignment against the Aiptasia genomic gene set database version 1.0 (Baumgarten et al., 2015), containing 29,269 entries. We also queried the phosphopeptides against the *B. minutum* genomic gene set (Aranda et al., 2016), which confirmed the presence of some *B. minutum* protein groups in symbiotic Aiptasia, the majority of those being notably absent in aposymbiotic samples (Figure S5, Dataset S5). The MaxQuant search output was imported into Spectronaut X  (Cat. Sw‐3001; Biognosys) for spectral library generation using default settings: minimum fragment ions length of three amino acids, fragment ions *m*/*z* range of 300–1,800, minimum relative intensity of 5%, and 3  to 6 fragment ions per precursor peptide. The DDA raw data and the spectral library are available via ProteomeXchange with identifier PXD014076.

### 2.9 DIA data processing

DIA raw data files were imported in Spectronaut X and the generated spectral library was assigned for identity retrieval of the quantified phosphopeptides, using default settings. Extraction and scoring of MS1 and MS2 mass tolerance as well as the retention time (RT) for the extracted ion chromatogram (XIC) were set to dynamic and correction factors of 1 were applied. Calibration of iRT standards was done automatically by local (non‐linear) regression. Mutated decoys (one to three random amino acids swap) were generated at a library size fraction of 0.1, a *q*‐value cutoff for precursor and protein identification was set to 0.01, and single protein hits were defined by stripped peptide sequences. Interference correction was applied so that the least interfering three fragment ions and two precursor ions were kept. All fragment ions not removed during interference correction were used for quantification, calculated from the area under the curve between the XIC boundaries of each targeted ion. Label‐free normalization was applied on the whole data set to minimize the effect of the potential variability generated by the sample preparation and the LC‐MS performance. Spectronaut X uses an algorithm based on the Local Regression Normalization described by Callister et al. (2006). This normalization approach assumes that the majority of the precursors in an experiment are not regulated (stable background), and when regulated, there is no preference for up‐ or down‐regulation. The *q*‐value sparse defines the normalization strategy; all precursors that passed the *q*‐value threshold at least in one sample across the experiment were used for normalization.

Upon completion of analysis, peptide precursor quantities (Fragment Group or FG) were exported, and a data matrix containing non‐redundant phosphopeptide precursors quantified across all samples (five aposymbiotic, five symbiotic) was generated for downstream analysis. The DIA raw data and the quantification file are available via ProteomeXchange with identifier PXD014076 (http://proteomecentral.proteomexchange.org/cgi/GetDataset?ID=PXD014076; https://www.ebi.ac.uk/pride/archive/projects/PXD014076).

### 2.10 Data analysis

Biological and statistical analysis of DDA and DIA data was performed in the Perseus environment (version 1.6.2.1) (Tyanova et al., 2016). The MaxQuant  search output of the 44 DDA files used to generate the phosphoproteome spectral library was imported in Perseus to retrieve the number of detected phosphosites, the ratio of phosphorylated amino acid residues, the phosphosite localization probabilities and the kinase motifs assignments. The phosphosite localization probabilities were assigned according to Olsen et al. (2006). That is, class I phosphosites were confidently assigned with probability above 0.75. Classes II and III had localization probabilities between 0.25 and 0.75, but to the former a matching kinase motif was assigned. No filtering based on phosphosite probability was made when generating the spectral library, because confident phosphosite assignment in the spectral library does not imply high probability assignment in the DIA data. As currently there is no control of correct phosphosite assignment for DIA in Spectronaut X, we relied on retention time shift of different phosphopeptides as well as manual validation of the spectra of interest.

The quantitative DIA data matrix covered 75% of the phosphopeptides compiled in the spectral library. The matrix was quality‐filtered so that only phosphopeptides with at least 70% of valid values across samples were considered; this reduced the phosphopeptide coverage to 62%. Prior to biological and statistical analysis, phosphopeptide quantities were log_2_‐transformed to achieve normal distribution. For principal component analysis (PCA) and Pearson's hierarchical clustering, missing values were inferred from a normal distribution. Briefly, Perseus shrinks the distribution to a width of 0.3 and down‐shifts the standard deviation by 1.8 times in order to mimic low phosphopeptide abundances, which are often the cause of missing values. Differentially abundant phosphopeptides across aposymbiotic and symbiotic conditions were statistically inferred by a two‐sided *t*‐test, with artificial within‐group variance S0 set to 0.1 (control of the *p*‐value, at S0 ≠ 0 the difference of means also plays a role), and multiple‐testing correction by permutation‐based FDR to .01 (250 randomizations without grouping preservation). For each protein‐annotated Aiptasia gene, KEGG orthology (KO) identifiers were retrieved in UniProtKB and visualized in KEGG Mapper. The annotation file for the KEGG enrichment was manually generated using the information from the KEGG BRITE tool, which provided classification of KOs into functional hierarchies. The KEGG enrichment analysis was performed by Fisher's exact test. Briefly, non‐random associations between differentially abundant phosphopeptides and KEGG processes were considered significant at *p* < .05. We set a relative enrichment parameter, which accounted for multiple phosphorylation sites on the same protein and considered each phosphoprotein only once in the enrichment analysis. Protein sequence alignments were performed with the multiple sequence alignment tool Clustal Omega (EMBL‐EBI) and regulatory functions for conserved phosphosites were searched in the PhosphoSitePlus database.

## 3 RESULTS

### 3.1 The Aiptasia phosphoproteome

**Figure 2:**
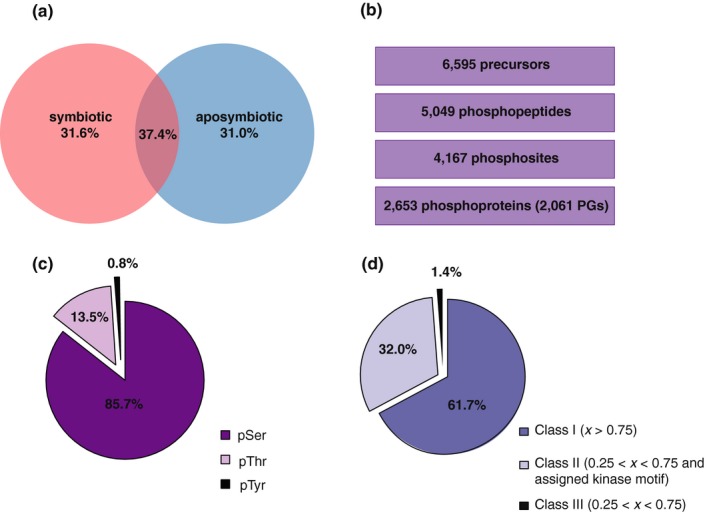
Phosphopeptide spectral library composition. (a) Aposymbiotic and symbiotic conditions contributed to the spectral library and were equally represented, as shown by the equal distribution of the protein groups (PGs). (b) Number of precursors, phosphopeptides, phosphosites and phosphoproteins (in PGs) encompassed by the library. (c) Relative presence of phosphorylated amino acid residues. (d) Localization probability (x) of the assigned phosphosites

To quantify protein phosphorylation in Aiptasia by DIA/SWATH‐MS, we first generated a phosphopeptide spectral library of highly fractionated aposymbiotic and symbiotic samples to increase coverage and detection of low abundant phosphopeptides. Aposymbiotic and symbiotic samples were equally represented in the spectral library (Figure 2a), which comprised 5,049 phosphopeptides (6,595 precursor ions), 4,167 phosphosites and 2,653 phosphoproteins clustering into 2,061 protein groups (PGs) (Figure 2b). When the phosphopeptide identity could not be confidently assigned due to the high sequence homology among phosphoproteins, these phosphoproteins clustered in the same PG. Phosphoproteins belonging to the same PG usually shared function, family, or presented redundant annotation. Phosphorylation occurred on the hydroxyl amino acid residues serine (Ser), threonine (Thr) and tyrosine (Tyr) at 85.7%, 13.5% and 0.8%, respectively (Figure 2c). Phosphorylation was confidently assigned to 61.7% of the amino acid residues (class I phosphosite, *p* > .75), whereas 33.4% of the phosphosites were assigned with a lower probability (.25 < *p* < .75) (Figure 2d). Nevertheless, for most of the low‐probability assignments a matching kinase binding motif was retrieved (class II phosphosites). Only in 1.4% of the assignments no phosphorylating kinase motif was identified (class III phosphosites).

### 3.2 Quantification of protein phosphorylation differences across symbiotic states

**Figure 3:**
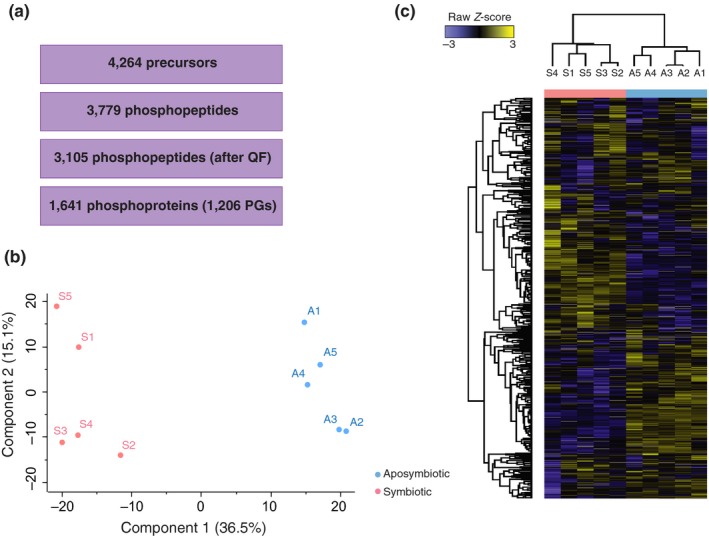
Quantification of protein phosphorylation across symbiotic conditions. (a) Number of precursor ions, phosphopeptides before and after quality filtering (QF), and phosphoproteins (grouped in PGs) consistently quantified across samples. (b) Principal component analysis (PCA) and (c) unsupervised Pearson's hierarchical clustering based on the quality filtered phosphoproteomic profiles of aposymbiotic (A, light blue) and symbiotic (S, pink) samples

We measured protein phosphorylation in aposymbiotic and symbiotic Aiptasia by DIA/SWATH‐MS and quantified 3,779 phosphopeptides, namely 75% of the phosphopeptides compiled in the spectral library (Figure 3a). For downstream analysis, we only retained phosphopeptides that were quantified in at least 70% of the experimental samples (i.e., present in at least 7 of the 10 samples). This reduced the matrix to 3,105 phosphopeptides (62% of the spectral library) and 1,641 phosphoproteins clustering into 1,206 PGs (Dataset S1). We quantified protein phosphorylation in aposymbiotic and symbiotic anemones based on phosphopeptide abundance and visualized sample clustering by PCA (Figure 3b). The PCA revealed clear sample separation based on symbiotic state (component 1, 36.5%), and the unsupervised Pearson's hierarchical clustering revealed characteristic phosphoproteomic profiles based on symbiotic conditions (Figure 3c), suggesting that protein phosphorylation differs between symbiotic states and might represent another level of “symbiotic control.” We next investigated which proteins and signalling pathways were targeted by differential phosphorylation.

### 3.3 Differential protein phosphorylation across symbiotic states group into distinct biological processes

**Figure 4:**
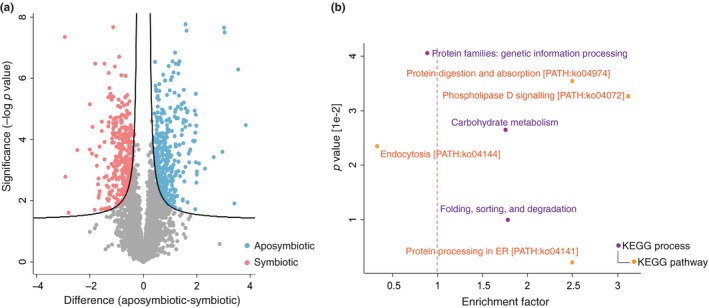
Differentially abundant phosphopeptides and enriched KEGG biological processes and signalling pathways. (a) Volcano plot representing highly abundant phosphopeptides in either symbiotic (pink, left side) or aposymbiotic (light blue, right side) Aiptasia anemones (FDR 0.01, S0 0.1). (b) Scatterplot representing KEGG processes and pathways targeted by differential phosphorylation (*p* < .05). KEGG terms are classified into processes (purple) and pathways (orange). Terms that are underrepresented in the analysis have an enrichment factor < 1 (left of the red dashed line)

Differential phosphorylation of proteins was statistically inferred by two‐sided *t*‐test (FDR 0.01, S0 0.1). The 644 differentially abundant phosphopeptides totalling 539 phosphoproteins (clustering into 390 PGs) were represented on a Volcano plot to illustrate highly abundant phosphopeptides in the symbiotic or aposymbiotic condition (Figure 4a and Dataset S1). To distinguish cases of differential phosphorylation from changes in phosphoprotein expression, we generated a total proteome data set from the same aposymbiotic and symbiotic replicates used for phosphoproteomic analysis (Figures S1 and S2, Dataset S3) and we normalized phosphopeptide amount against total protein abundance. This was done for all the differentially phosphorylated proteins highlighted by the KEGG enrichment analysis (Figure 4b). According to the KEGG enrichment analysis, among the biological processes and signalling pathways that were differentially phosphorylated  (*p* < .05), we found “carbohydrate metabolism,” “protein digestion and absorption (ko04974),” “phospholipase D signalling pathway (ko04072),” “protein folding, sorting and degradation” and “protein processing in endoplasmic reticulum (ER) (ko04141)” (Dataset S2). Nevertheless, the total proteome normalization revealed that differential phosphorylation of proteins belonging to the KEGG terms “carbohydrate metabolism” and “protein digestion and absorption” co‐occurred with changes in the total amount of these respective proteins. Examples were key metabolic enzymes (e.g., ATP‐citrate synthase, AIPGENE6154; acetyl‐CoA carboxylase, AIPGENE11605; long‐chain‐fatty‐acid‐CoA ligase 1, AIPGENE7314) and amino acid transporters (e.g., b(0,+)‐type amino acid transporter 1, AIPGENE1716 and AIPGENE15008; rBAT, AIPGENE 7824). Because discrimination between translational and post‐translational changes is critical for the inference of biological regulation, we focused on the KEGG terms that were target of differential phosphorylation at constant protein abundance.

### 3.4 The phospholipase D signalling pathway

**Figure 5:**
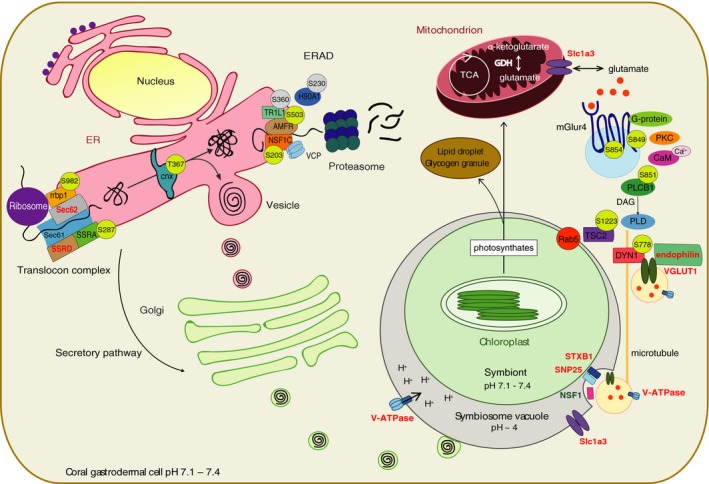
Model of a symbiotic cnidarian cell representing putative phosphorylation‐mediated regulation of the KEGG terms “PLD signalling pathway” and “protein processing in ER.” For each differentially phosphorylated protein, the phosphosite is represented in a circle as phosphorylated (yellow) or dephosphorylated (grey) in symbiotic versus aposymbiotic Aiptasia. For the sake of completeness, the model includes additional proteins that belong to the two pathways. Bold font indicates proteins that were measured but not differentially abundant. The colored font represents upregulation (red) and downregulation (green) of protein abundance in symbiosis

In the KEGG enrichment analysis, the “phospholipase D signalling pathway (ko04072)” was highly enriched (enrichment factor 3.12), and thus targeted by differential phosphorylation in symbiosis. The phospholipase D (PLD) signalling pathway is activated by the binding of G protein‐coupled receptors (GPCRs) to their ligands. The metabotropic glutamate receptors (mGluRs) are glutamate‐binding GPCRs that trigger the PLD signalling cascade via phospholipase C (PLC)‐induced Ca^2+^ mobilization and protein kinase C (PKC) activation (Figure 5). In Aiptasia, the mGluR family subtype mGluR4 (AIPGENE24030; AIPGENE24031) was highly phosphorylated at its carboxyl terminus in symbiotic versus aposymbiotic anemones. Phosphorylation occurred at Ser849‐p and Ser854‐p, in the predicted cytosolic terminus following the 7th transmembrane domain. In silico kinase motif assignment indicated that Ser849‐p but not Ser854‐p on Aiptasia mGluR4 is a substrate for PKC‐mediated phosphorylation. Downstream of mGluR4, the PLC subunit β1 (PLCβ1, AIPGENE26754) was also highly phosphorylated at Ser851‐p in symbiosis.

The PLD pathway cross‐regulates multiple signalling cascades horizontally and vertically. We found differential phosphorylation of effector molecules belonging to various PLD‐interacting pathways. Tuberin (TSC2, AIPGENE9407) is a regulator of the mTOR pathway that also has a role in microtubule‐mediated protein transport and in the regulation of the GTPase activity of the Ras‐related protein Rab5. TSC2 was highly phosphorylated at Ser1223‐p in symbiosis. Dynamin‐1 (DYN1, AIPGENE26789), a microtubule‐associated phosphoprotein involved in vesicular trafficking, was highly phosphorylated in symbiosis at Ser778‐p, which is a conserved phosphosite in proximity to the mammalian regulatory phosphosite Ser774‐p (not present in Aiptasia). Additionally, our proteome data set showed enrichment of the process “nervous system” (Figure S4b and Dataset S4), which relates to the synaptic cycling of glutamate vesicles. Indeed, together with a 0.91‐fold upregulation in symbiosis of VGLUT1 (AIPGENE24249), we found a 0.54‐fold upregulation of endophilin (AIPGENE20180), a 0.69‐fold upregulation of the glutamate transporter Slc1a3 (AIPGENE25062), a 0.73‐fold upregulation of the vacuolar‐type H^+^ ATPase (V‐ATPase, AIPGENE26700) and differential regulation of several components of the large SNARE protein complex (STXB1, AIPGENE18555, +0.42‐fold; SNP25, AIPGENE14343, +0.49‐fold; NSF1, AIPGENE23965, −0.54‐fold) (Dataset S3), which is responsible for vesicle docking and fusion, as well as glutamate release.

### 3.5 Protein processing in the endoplasmic reticulum

Another highly enriched pathway (enrichment factor 2.50) in the KEGG analysis was “protein processing in ER (ko04141),” a pathway that belongs to the higher order process “folding, sorting and degradation,” which was also slightly enriched (enrichment factor 1.78). Interestingly, the process “folding, sorting and degradation” (Figure 4b) was enriched at the phosphoproteome and under‐represented (enrichment factor < 1) at the proteome level (Figure S3b and Dataset S4), suggesting that regulation of this process occurs post‐translationally. Indeed, in the endoplasmic reticulum (ER) of symbiotic Aiptasia, we found higher phosphorylation of several components of the translocon, a protein complex responsible for the import of proteins from their site of synthesis in cytosolic ribosomes into the ER lumen. In particular, we found higher phosphorylation of the ribosome‐binding protein 1 at Ser982‐p (rrbp1, AIPGENE 7803) and of the translocon‐associated protein (TRAP) subunit α (SSRA, AIPGENE11225) at Ser287‐p (Figure 5). Phosphorylation of TRAP was also coupled with higher expression (+0.47‐fold) of its other subunit *δ* (SSRD, AIPGENE5652) and of the translocation protein SEC62 (AIPGENE12061, +0.49‐fold). Higher phosphorylation was also observed at Thr367‐p on the lectin‐type chaperone calnexin (cnx, AIPGENE23380), a Ca^2+^‐binding protein with a major function in the quality control of protein folding. Properly folded proteins are sorted to the Golgi (secretory pathway), whereas misfolded proteins undergo ER‐associated degradation (ERAD). Across symbiotic states, we found substantial differential phosphorylation of the ERAD apparatus. For instance, we found that dephosphorylation of the E3 ubiquitin‐protein ligase AMFR (AMFR, AIPGENE14825) at Ser503‐p. AMFR is part of the VCP/p97‐AMFR/gp78 complex, which is involved in the final step of ERAD. The NSFL1 cofactor p47 (NSF1C, AIPGENE8167), a regulator of the ATPase activity of the above‐mentioned VCP (transitional endoplasmic reticulum ATPase), was highly phosphorylated on Ser203‐p, whereas the translocating chain‐associated membrane protein 1‐like 1 (TR1L1, AIPGENE17645) and the heat shock protein HSP 90‐alpha 1 (H90A1, AIPGENE3199) were dephosphorylated on Ser360‐p and Ser230‐p, respectively.

## 4 DISCUSSION

Our work represents the first phosphoproteome generated for a cnidarian–algal symbiotic association as well as the first application of label‐free phosphoproteomic quantification by DIA/SWATH‐MS in the coral field. As such, we focused on the generation of a comprehensive phosphopeptide spectral library for Aiptasia, namely a collection of phosphopeptide spectra that serves as a reference database for phosphopeptide identification during quantification in DIA/SWATH‐MS. The generation of a spectral library that equally represents the aposymbiotic and symbiotic states (Figure 2a) was relevant to avoid quantification biases deriving from heterogeneous phosphopeptide representation. The ratio of phosphorylated amino acid residues (Figure 2c) was comparable to that found in other organisms (Olsen et al., 2006; Robles, Humphrey, & Mann, 2017), and in most of the cases, phosphosites were confidently assigned or identified as a substrate of known protein kinases (Figure 2d). Quantification in DIA/SWATH‐MS revealed that the phosphoproteomic profile of the samples reflected their symbiotic state (Figure 3b,c). Of the 390 differentially phosphorylated PGs (Figure 4a), the enrichment analysis showed that only 84 clustered into KEGG terms and therefore contributed to the enrichment. This suggests that differential phosphorylation occurs across a broad range of proteins rather than pre‐dominantely targeting selected biological processes or signalling pathways. Here we focused on the differentially phosphorylated proteins highlighted by the enrichment analysis in order to describe potential signalling pathways associated with coral–algal symbiosis.

The differential abundance of a measured phosphopeptide can result either from changes in phosphorylation at a specific phosphosite or from variation in the translated protein (at constant phosphosite occupancy or stoichiometry), or a combination of the two events (Wu et al., 2011). Among the phospho‐regulated proteins, we promptly  recognized some for which regulation at the level of protein expression during symbiosis was previously described (Oakley et al., 2016). In such cases, differential phosphopeptide abundance probably reflected an increase in total phosphoprotein amount (by de novo protein synthesis) rather than differential phosphorylation. Although such cases did not allow for *sensu stricto* discrimination between translational and post‐translational change, the presence of phosphoproteins belonging to the KEGG terms “carbohydrate metabolism” and “protein digestion and absorption” represented a “sanity check” of our data set. This is because genes/proteins associated with “carbohydrate metabolism” and “protein digestion and absorption” (or amino acid transport) have been characterized at the transcriptional/translational level in symbiotic cnidarians and showed adjustment to the availability of photosynthetically fixed carbon translocated by the endosymbiotic algae as well as increased amino acid synthesis and transport (possibly to the algae for primary production) (Kopp et al., 2015; Lehnert et al., 2014; Oakley et al., 2016; Rädecker et al., 2015). Here we suggest that proteins associated with these processes are targeted by phosphorylation in cnidarians. Indeed,  some phosphosites on these proteins are conserved and have known regulatory function in higher vertebrates (Berg, Tymoczko, Stryer, & Clarke, 2002; Frahm, Li, Grevengoed, & Coleman, 2011; Ray, Suau, Vincent, & Dalla Venezia, 2009), suggesting their possible relevance also in Aiptasia.

Among the KEGG pathways that were differentially phosphorylated (at constant protein abundance) in symbiosis, we found the “PLD signalling pathway”. To our knowledge, this pathway has not been previously described in the context of the coral–algal symbiosis. The PLD pathway is a signal transduction cascade regulated mainly by phosphorylation and regulates a variety of symbiotic processes, including cytoskeletal reorganization, intracellular vesicle trafficking and receptor‐mediated endo‐/phagocytosis, commonly described in the coral–algal symbiosis (Baumgarten et al., 2015; Davy et al., 2012; Li et al., 2018). Amongst these processes, the PLD pathway regulates vesicle trafficking across intracellular compartments (Cazzolli, Shemon, Fang, & Hughes, 2006; Jones, Morgan, & Cockcroft, 1999), namely the intracellular transport of cargo molecules (e.g., amino acids, proteins, lipids) mediated by vesicular carriers.

The PLD pathway can be activated by the metabotropic glutamate receptor mGluR4 that self‐phosphorylates upon glutamate binding. In symbiosis, we found higher phosphorylation of mGluR4 at phosphosite Ser849‐p, a potential substrate for PKC‐mediated phosphorylation and a binding site for Ca^2+^/calmodulin (CaM) (Figure 5). Based on sequence alignment, this phosphosite resides in a sequence stretch that is not present in the mGluR4 of higher vertebrates. However, mGluR4 is closely related to mGluR7, the most conserved mGluR subtype in mammals (Nakajima, Yamamoto, Nakayama, & Nakanishi, 1999). Interestingly, not only is Ser849‐p conserved between Aiptasia mGluR4 and the mammalian mGluR7, but also phosphorylation at this phosphosite in mGluR7 is mediated by PKC and inhibited by the binding of Ca^2+^/CaM (Nakajima et al., 1999). This suggests that similarly to mGluR7 in higher vertebrates, mGluR4 in Aiptasia may regulate the PLD signalling transduction cascade in a PKC/Ca^2+^/CaM/PLC‐dependent manner. The phospholipase PLCβ1 was indeed also highly phosphorylated in symbiosis. In higher vertebrates, PLCβ1 can be phosphorylated at multiple sites, although only phosphorylation at Ser982‐p has yet been shown to positively regulate its enzymatic activity, and thus the formation of the second messenger molecules diacylglycerol (DAG) and inositol 1,4,5‐trisphosphate (IP3) (Xu et al., 2001). Interestingly, PLC and its reaction products, DAG and IP3, are also involved in the “phosphatidylinositol signalling system,” process that was previously described in the regulation of endocytosis in other symbiotic systems and implied in the modulation of the coral–algal interaction (Peleg‐Grossman, Volpin, & Levine, 2007; Rosic et al., 2014).

In the symbiotic state, we also found phosphorylation of effector proteins belonging to interacting pathways downstream of PLD. An example is the mTOR modulator TSC2, which also regulates the GTPase activity of Rab5 (Chen, Cheng, Hong, & Fang, 2004; Jiang & Yeung, 2006; Xiao, Shoarinejad, Jin, Golemis, & Yeung, 1997). Rab5 is an important modulator of endo‐/phagocytosis and was previously shown to localize at the symbiosome in Aiptasia, potentially conferring the characteristics of an arrested phagosome (Chen et al., 2004; Dani et al., 2017; Venn et al., 2009). The microtubule‐associated protein DYN1 was phosphorylated near the mammalian phosphosite Ser774‐p. Richter et al. (2018) recently showed that phosphorylation at Ser774‐p modulates the binding of DYN1 to the vesicular glutamate transporter 1 (VGLUT1) and to endophilin, hence regulating the intracellular trafficking of glutamate‐containing vesicles. Intriguingly, our total proteome data set revealed upregulation of both endophilin and VGLUT1, as well as differential expression of several components of the large SNARE protein complex that has a major function in vesicular trafficking and glutamate release. Together, these findings suggest that in the cnidarian–algal symbiosis the PLD signalling pathway may be activated by the binding of glutamate to its receptor mGluR4, triggering a downstream phosphorylation cascade that regulates vesicular trafficking across symbiotic compartments. It was shown that mGlur4 could localize on cytoplasmic vesicles (Iacovelli et al., 2004), perhaps sensing host cytosolic glutamate concentrations and activating its transport to the symbiosome. This would imply that glutamate regulates its transport inside VGLUT1 vesicles across symbiotic compartments, thus reconfirming its importance in the recycling of nitrogen and modulation of the cnidarian–algal symbiotic interaction, especially given the nitrogen‐poor state of reef waters (Macdonald, Lin, Russell, Thomas, & Douglas, 2012).

Another pathway that was differentially phosphorylated (at constant protein abundance) in symbiosis was “protein processing in ER.” Here we found higher phosphorylation of some components (rrbp1, SSRA) of the translocon, the protein complex responsible for the import of newly synthesized proteins from cytosolic ribosomes into the ER (Figure 5). Phosphorylation occurred together with the upregulation of some other translocon components (SSRD, Sec62), as well as phosphorylation of the lectin‐type chaperone calnexin, a key player in the control of protein fate (Bergeron, Brenner, Thomas, & Williams, 1994). Also, several components of ERAD (AMFR, NSF1C, TR1L1, H90A1) were differentially phosphorylated in symbiosis. Notably, Ser503‐p of AMFR is conserved in higher vertebrates (corresponding to Ser522‐p). In human cell lines, AMFR phosphorylation at a nearby site (Ser516‐p) triggers its ubiquitination and degradation (Wang et al., 2018). Indeed, while properly folded proteins leave the ER via the secretory pathway (i.e., proteins are packed into vesicles and delivered to the Golgi for further distribution to target compartments), misfolded proteins are tagged for ubiquitination in the ER and retrotranslocated to cytosolic proteasomes for degradation via the ERAD apparatus (Fumagalli et al., 2016). The coordinated phosphorylation and upregulation of different components of the ER machinery during symbiosis may indicate sustained import of newly synthesized proteins in the ER for folding and delivery to other compartments via the secretory pathway, as well as high protein turnover via degradation.

Differential regulation of the ER machinery in the cnidarian–algal symbiosis has not been extensively described before, in agreement with the notion that we detected enrichment at the post‐translational but not at the translational level. Oakley et al. (2017) showed proteostasis disruption and ER stress in Aiptasia under thermal‐shock, suggesting that damage of this compartment in cnidarians may be involved in symbiosis breakdown. Additionally, none of the identified phosphosites has been functionally characterized in other organisms, and therefore no direction of regulation could be inferred in our system. We found some literature on the relevance of the protein secretory pathway (ER, Golgi and vesicle trafficking) in symbiotic metazoans and plants (Wang & Dong, 2011; Wang et al., 2010). This pathway is highly conserved among eukaryotes, and sessile organisms such as plants rely on the secretory pathway to adjust to changing environmental conditions, avoid pathogen infection, and achieve symbiosis (Wang & Dong, 2011). Intriguingly, in plant–root microbial symbioses the secretory pathway is used by the host to deliver proteins to the symbiosome (or periarbuscolar) membrane. Although not strictly secreted, these proteins would still be processed in the ER and Golgi, packed into vesicles and directed to the symbiosome via cytoskeleton and associated motor proteins (Wang & Dong, 2011). In concert, regulation of multiple components of the secretory pathway suggests its essential function in maintaining the complex mosaic of proteins that decorate the symbiosome (Maunoury et al., 2010; Pumplin & Harrison, 2009; Wang et al., 2010). In the cnidarian–algal symbiosis, the differential phosphorylation of various components of the ER machinery and its implication in protein translocation, folding and degradation could suggest a potentially important regulatory function of this compartment in the recycling and degradation of the large suite of proteins necessary to maintain the symbiosome as an arrested phagosome.

Overall our findings highlight changes in the phosphorylation status of PLD signalling and of protein processing in the ER, suggesting that phosphorylation may be a relevant regulatory mechanism of these pathways during an established cnidarian–algal interaction. This supports the concept of “dynamic stability” of the cnidarian–algal association (Davy et al., 2012). This dynamism may reflect a system in continuous readjustment with possible implications for the stability of the association, which can be disrupted at every stage if no longer fulfilling the requirements of the symbiotic partners.

Clearly, all our findings need further functional validation. Because protein phosphorylation is a reversible modification, time‐series measurements under system perturbation, such as recolonization of the host with one or more algal strains or induction of symbiosis breakdown, would show how the phosphorylation status changes and point towards a direction of regulation. Additionally, the establishment of an Aiptasia or coral endodermal cell line would further allow characterization of protein phosphorylation by means of chemical inhibition of kinases or phosphatases that specifically phospho‐/dephosphorylate proteins of interest. Ultimately, functional validation of phosphorylation by phosphosite‐directed mutagenesis thanks to emerging genome editing techniques in the field (Cleves, Strader, Bay, Pringle, & Matz, 2018) could unveil regulatory phosphosites.

## 5 CONCLUSIONS

This is the first phosphoproteomic study of a cnidarian–algal symbiotic association as well as the first application of label‐free quantification by DIA/SWATH‐MS in the field. The phosphopeptide spectral library is a publicly available resource for cross‐laboratory quantification studies and the quantitative data set can be used to explore regulation of more than 1,000 phosphoproteins in the cnidarian–algal symbiosis. Quantification of protein phosphorylation in aposymbiotic and symbiotic Aiptasia revealed changes in the phosphorylation status of the PLD signalling pathway and protein processing in the ER, revealing for the first time a putative layer of post‐translational regulation in a cnidarian–algal symbiotic interaction. We anticipate that the establishment of the DIA/SWATH‐MS method in the field will pave the way  to further studies and more sophisticated experimental designs and will help addressing new biological questions in the context of the cnidarian–algal symbiosis.

## AUTHOR CONTRIBUTIONS

F.S. and C.R.V. conceived the study and designed the experiments; F.S. and H.Z. performed the experiments and processed the data; F.S. analysed the data; F.S. and C.R.V. wrote the manuscript with contributions from H.Z. All authors read and approved the final manuscript.

## Data Availability

The MS proteomics data (DDA and DIA raw files, phosphopeptide spectral library, quantification files) have been deposited with the ProteomeXchange Consortium via the PRIDE partner repository (Perez‐Riverol et al., 2019) with the data set identifier PXD014076 (http://proteomecentral.proteomexchange.org/cgi/GetDataset?ID=PXD014076; https://www.ebi.ac.uk/pride/archive/projects/PXD014076).

## References

[mec15298-bib-0001] Aivaliotis, M. , Macek, B. , Gnad, F. , Reichelt, P. , Mann, M. , & Oesterhelt, D. (2009). Ser/Thr/Tyr protein phosphorylation in the archaeon *Halobacterium salinarum*—A representative of the third domain of life. PLoS ONE, 4, e4777 10.1371/journal.pone.0004777 19274099 PMC2652253

[mec15298-bib-0002] Aranda, M. , Li, Y. , Liew, Y. J. , Baumgarten, S. , Simakov, O. , Wilson, M. C. , … Voolstra, C. R. (2016). Genomes of coral dinoflagellate symbionts highlight evolutionary adaptations conducive to a symbiotic lifestyle. Scientific Reports, 6, 39734 10.1038/srep39734 28004835 PMC5177918

[mec15298-bib-0003] Batth, T. S. , & Olsen, J. V. (2016). Offline high pH reversed‐phase peptide fractionation for deep phosphoproteome coverage. Methods in Molecular Biology (Clifton, NJ), 1355, 179–192. 10.1007/978-1-4939-3049-4_12 26584926

[mec15298-bib-0004] Baumgarten, S. , Simakov, O. , Esherick, L. Y. , Liew, Y. J. , Lehnert, E. M. , Michell, C. T. , … Voolstra, C. R. (2015). The genome of Aiptasia, a sea anemone model for coral symbiosis. Proceedings of the National Academy of Sciences of the United States of America, 112, 11893–11898. 26324906 10.1073/pnas.1513318112 PMC4586855

[mec15298-bib-0005] Berg, J. M. , Tymoczko, J. L. , Stryer, L. , & Clarke, N. D. (2002). Section 22.5 acetyl coenzyme a carboxylase plays a key role in controlling fatty acid metabolism (5th ed.). New York, NY: W. H. Freeman.

[mec15298-bib-0006] Bergeron, J. J. M. , Brenner, M. B. , Thomas, D. Y. , & Williams, D. B. (1994). Calnexin: A membrane‐bound chaperone of the endoplasmic reticulum. Trends in Biochemical Sciences, 19, 124–128. 10.1016/0968-0004(94)90205-4 8203019

[mec15298-bib-0007] Callister, S. J. , Barry, R. C. , Adkins, J. N. , Johnson, E. T. , Qian, W. J. , Webb‐Robertson, B. J. , … Lipton, M. S. (2006). Normalization approaches for removing systematic biases associated with mass spectrometry and label‐free proteomics. Journal of Proteome Research, 5, 277–286. 10.1021/pr050300l 16457593 PMC1992440

[mec15298-bib-0008] Cazzolli, R. , Shemon, A. N. , Fang, M. Q. , & Hughes, W. E. (2006). Phospholipid signalling through phospholipase D and phosphatidic acid. IUBMB Life, 58, 457–461. 10.1080/15216540600871142 16916782

[mec15298-bib-0009] Chen, M. C. , Cheng, Y. M. , Hong, M. C. , & Fang, L. S. (2004). Molecular cloning of Rab5 (ApRab5) in *Aiptasia pulchella* and its retention in phagosomes harboring live zooxanthellae. Biochemical and Biophysical Research Communications, 324, 1024–1033. 10.1016/j.bbrc.2004.09.151 15485657

[mec15298-bib-0010] Cleves, P. A. , Strader, M. E. , Bay, L. K. , Pringle, J. R. , & Matz, M. V. (2018). CRISPR/Cas9‐mediated genome editing in a reef‐building coral. Proceedings of the National Academy of Sciences of the United States of America, 115, 5235–5240. 10.1073/pnas.1722151115 29695630 PMC5960312

[mec15298-bib-0011] Cozzone, A. J. (2005). Role of protein phosphorylation on serine/threonine and tyrosine in the virulence of bacterial pathogens. Journal of Molecular Microbiology and Biotechnology, 9, 198–213. 10.1159/000089648 16415593

[mec15298-bib-0012] Dani, V. , Priouzeau, F. , Mertz, M. , Mondin, M. , Pagnotta, S. , Lacas‐Gervais, S. , … Sabourault, C. (2017). Expression patterns of sterol transporters NPC1 and NPC2 in the cnidarian‐dinoflagellate symbiosis. Cellular Microbiology, 19(10), e12753 10.1111/cmi.12753 28544363

[mec15298-bib-0013] Davy, S. K. , Allemand, D. , & Weis, V. M. (2012). Cell biology of cnidarian‐dinoflagellate symbiosis. Microbiology and Molecular Biology Reviews: MMBR, 76, 229–261. 10.1128/MMBR.05014-11 22688813 PMC3372257

[mec15298-bib-0014] Davy, S. K. , Lucas, I. A. N. , & Turner, J. R. (1996). Carbon budgets in temperate anthozoan‐dinoflagellate symbioses. Marine Biology, 126, 773–783. 10.1007/BF00351344

[mec15298-bib-0015] Dubinsky, Z. , & Stambler, N. (2010). Coral reefs: An ecosystem in transition. Berlin, Germany: Springer Science & Business Media.

[mec15298-bib-0016] Engholm‐Keller, K. , Birck, P. , Størling, J. , Pociot, F. , Mandrup‐Poulsen, T. , & Larsen, M. R. (2012). TiSH — A robust and sensitive global phosphoproteomics strategy employing a combination of TiO_2_, SIMAC, and HILIC. Journal of Proteomics, 75, 5749–5761. 10.1016/j.jprot.2012.08.007 22906719

[mec15298-bib-0017] Enríquez, S. , Méndez, E. R. , & Prieto, R. I. (2005). Multiple scattering on coral skeletons enhances light absorption by symbiotic algae. Limnology and Oceanography, 50, 1025–1032. 10.4319/lo.2005.50.4.1025

[mec15298-bib-0018] Falkowski, P. G. , Dubinsky, Z. , Muscatine, L. , & Porter, J. W. (1984). Light and the bioenergetics of a symbiotic coral. BioScience, 34, 705–709. 10.2307/1309663

[mec15298-bib-0019] Frahm, J. L. , Li, L. O. , Grevengoed, T. J. , & Coleman, R. A. (2011). Phosphorylation and acetylation of Acyl‐CoA synthetase‐I. Journal of Proteomics & Bioinformatics, 4, 129–137. 10.4172/jpb.1000180 24039348 PMC3772793

[mec15298-bib-0020] Fumagalli, F. , Noack, J. , Bergmann, T. J. , Cebollero, E. , Pisoni, G. B. , Fasana, E. , … Molinari, M. (2016). Translocon component Sec62 acts in endoplasmic reticulum turnover during stress recovery. Nature Cell Biology, 18, 1173–1184. 10.1038/ncb3423 27749824

[mec15298-bib-0021] Ganot, P. , Moya, A. , Magnone, V. , Allemand, D. , Furla, P. , & Sabourault, C. (2011). Adaptations to endosymbiosis in a cnidarian‐dinoflagellate association: Differential gene expression and specific gene duplications. PLoS Genetics, 7, e1002187 10.1371/journal.pgen.1002187 21811417 PMC3141003

[mec15298-bib-0022] Hoegh‐Guldberg, O. , Mumby, P. J. , Hooten, A. J. , Steneck, R. S. , Greenfield, P. , Gomez, E. , … Hatziolos, M. E. (2007). Coral reefs under rapid climate change and ocean acidification. Science, 318, 1737–1742. 10.1126/science.1152509 18079392

[mec15298-bib-0023] Humphrey, S. J. , James, D. E. , & Mann, M. (2015). Protein phosphorylation: A major switch mechanism for metabolic regulation. Trends in Endocrinology & Metabolism, 26, 676–687. 10.1016/j.tem.2015.09.013 26498855

[mec15298-bib-0024] Iacovelli, L. , Capobianco, L. , Iula, M. , Di Giorgi Gerevini, V. , Picascia, A. , Blahos, J. , … De Blasi, A. (2004). Regulation of mGlu4 metabotropic glutamate receptor signaling by type‐2 G‐protein coupled receptor kinase (GRK2). Molecular Pharmacology, 65, 1103–1110. 10.1124/mol.65.5.1103 15102938

[mec15298-bib-0025] Imbs, A. B. (2013). Fatty acids and other lipids of corals: Composition, distribution, and biosynthesis. Russian Journal of Marine Biology, 39(3), 153–168. 10.1134/S1063074013030061

[mec15298-bib-0026] Jaspers, C. , Fraune, S. , Arnold, A. E. , Miller, D. J. , Bosch, T. C. G. , & Voolstra, C. R. (2019). Resolving structure and function of metaorganisms through a holistic framework combining reductionist and integrative approaches. Zoology, 133, 81–87. 10.1016/j.zool.2019.02.007 30979392

[mec15298-bib-0027] Jiang, X. , & Yeung, R. S. (2006). Regulation of microtubule‐dependent protein transport by the TSC2/mammalian target of rapamycin pathway. Cancer Research, 66, 5258–5269. 10.1158/0008-5472.CAN-05-4510 16707451

[mec15298-bib-0028] Jones, D. , Morgan, C. , & Cockcroft, S. (1999). Phospholipase D and membrane traffic: Potential roles in regulated exocytosis, membrane delivery and vesicle budding. Biochimica Et Biophysica Acta (BBA) – Molecular and Cell Biology of Lipids, 1439, 229–244. 10.1016/S1388-1981(99)00097-9 10425398

[mec15298-bib-0029] Jünger, M. A. , & Aebersold, R. (2014). Mass spectrometry‐driven phosphoproteomics: Patterning the systems biology mosaic. Wiley Interdisciplinary Reviews: Developmental Biology, 3, 83–112. 10.1002/wdev.121 24902836

[mec15298-bib-0030] Kopp, C. , Domart‐Coulon, I. , Escrig, S. , Humbel, B. M. , Hignette, M. , & Meibom, A. (2015). Subcellular investigation of photosynthesis‐driven carbon assimilation in the symbiotic reef coral *Pocillopora damicornis* . MBio, 6(1), e02299‐14 10.1128/mBio.02299-14 25670779 PMC4337570

[mec15298-bib-0031] LaJeunesse, T. C. , Parkinson, J. E. , Gabrielson, P. W. , Jeong, H. J. , Reimer, J. D. , Voolstra, C. R. , & Santos, S. R. (2018). Systematic revision of Symbiodiniaceae highlights the antiquity and diversity of coral endosymbionts. Current Biology, 28, 2570–2580.e6. 10.1016/j.cub.2018.07.008 30100341

[mec15298-bib-0032] Lehnert, E. M. , Burriesci, M. S. , & Pringle, J. R. (2012). Developing the anemone Aiptasia as a tractable model for cnidarian‐dinoflagellate symbiosis: The transcriptome of aposymbiotic *A. pallida* . BMC Genomics, 13, 271 10.1186/1471-2164-13-271 22726260 PMC3427133

[mec15298-bib-0033] Lehnert, E. M. , Mouchka, M. E. , Burriesci, M. S. , Gallo, N. D. , Schwarz, J. A. , & Pringle, J. R. (2014). Extensive differences in gene expression between symbiotic and aposymbiotic cnidarians. G3 – Genes|genomes|genetics, 4(2), 277–295. 10.1534/g3.113.009084 24368779 PMC3931562

[mec15298-bib-0034] Li, Y. , Liew, Y. J. , Cui, G. , Cziesielski, M. J. , Zahran, N. , Michell, C. T. , … Aranda, M. (2018). DNA methylation regulates transcriptional homeostasis of algal endosymbiosis in the coral model Aiptasia. Science Advances, 4(8), eaat2142 10.1126/sciadv.aat2142 30116782 PMC6093633

[mec15298-bib-0035] Macdonald, S. J. , Lin, G. G. , Russell, C. W. , Thomas, G. H. , & Douglas, A. E. (2012). The central role of the host cell in symbiotic nitrogen metabolism. Proceedings of the Royal Society B: Biological Sciences, 279, 2965–2973. 10.1098/rspb.2012.0414 PMC3385485 22513857

[mec15298-bib-0036] Matthews, J. L. , Crowder, C. M. , Oakley, C. A. , Lutz, A. , Roessner, U. , Meyer, E. , … Davy, S. K. (2017). Optimal nutrient exchange and immune responses operate in partner specificity in the cnidarian‐dinoflagellate symbiosis. Proceedings of the National Academy of Sciences of the United States of America, 114, 13194–13199. 10.1073/pnas.1710733114 29158383 PMC5740609

[mec15298-bib-0037] Maunoury, N. , Redondo‐Nieto, M. , Bourcy, M. , Van de Velde, W. , Alunni, B. , Laporte, P. , … Mergaert, P. (2010). Differentiation of symbiotic cells and endosymbionts in *Medicago truncatula* nodulation are coupled to two transcriptome‐switches. PLoS ONE, 5, e9519 10.1371/journal.pone.0009519 20209049 PMC2832008

[mec15298-bib-0038] Muscatine, L. , Falkowski, P. , Porter, J. , & Dubinsky, Z. (1984). Fate of photosynthetic fixed carbon in light‐and shade‐adapted colonies of the symbiotic coral *Stylophora pistillata* . Proceedings of the Royal Society of London Series B Biological Sciences, 222, 181–202.

[mec15298-bib-0039] Nakajima, Y. , Yamamoto, T. , Nakayama, T. , & Nakanishi, S. (1999). A relationship between protein kinase C phosphorylation and calmodulin binding to the metabotropic glutamate receptor subtype 7. The Journal of Biological Chemistry, 274, 27573–27577. 10.1074/jbc.274.39.27573 10488094

[mec15298-bib-0040] Nielsen, D. A. , Petrou, K. , & Gates, R. D. (2018). Coral bleaching from a single cell perspective. The ISME Journal, 12, 1558–1567. 10.1038/s41396-018-0080-6 29463894 PMC5955907

[mec15298-bib-0041] Oakley, C. A. , Ameismeier, M. F. , Peng, L. , Weis, V. M. , Grossman, A. R. , & Davy, S. K. (2016). Symbiosis induces widespread changes in the proteome of the model cnidarian Aiptasia. Cellular Microbiology, 18, 1009–1023. 26716757 10.1111/cmi.12564

[mec15298-bib-0042] Oakley, C. A. , Durand, E. , Wilkinson, S. P. , Peng, L. , Weis, V. M. , Grossman, A. R. , & Davy, S. K. (2017). Thermal shock induces host proteostasis disruption and endoplasmic reticulum stress in the model symbiotic cnidarian Aiptasia. Journal of Proteome Research, 16, 2121–2134. 28474894 10.1021/acs.jproteome.6b00797

[mec15298-bib-0043] Ochoa, D. , Jonikas, M. , Lawrence, R. T. , El Debs, B. , Selkrig, J. , Typas, A. , … Beltrao, P. (2016). An atlas of human kinase regulation. Molecular Systems Biology, 12, 888 10.15252/msb.20167295 27909043 PMC5199121

[mec15298-bib-0044] Olsen, J. V. , Blagoev, B. , Gnad, F. , Macek, B. , Kumar, C. , Mortensen, P. , & Mann, M. (2006). Global, in vivo, and site‐specific phosphorylation dynamics in signaling networks. Cell, 127, 635–648. 10.1016/j.cell.2006.09.026 17081983

[mec15298-bib-0045] Peleg‐Grossman, S. , Volpin, H. , & Levine, A. (2007). Root hair curling and Rhizobium infection in *Medicago truncatula* are mediated by phosphatidylinositide‐regulated endocytosis and reactive oxygen species. Journal of Experimental Botany, 58, 1637–1649. 10.1093/jxb/erm013 17420174

[mec15298-bib-0046] Perez‐Riverol, Y. , Csordas, A. , Bai, J. , Bernal‐Llinares, M. , Hewapathirana, S. , Kundu, D. J. , … Vizcaíno, J. A. (2019). The PRIDE database and related tools and resources in 2019: Improving support for quantification data. Nucleic Acids Research, 47, D442–D450. 10.1093/nar/gky1106 30395289 PMC6323896

[mec15298-bib-0047] Pumplin, N. , & Harrison, M. J. (2009). Live‐cell imaging reveals periarbuscular membrane domains and organelle location in *Medicago truncatula* roots during arbuscular mycorrhizal symbiosis. Plant Physiology, 151, 809–819. 19692536 10.1104/pp.109.141879 PMC2754618

[mec15298-bib-0048] Rädecker, N. , Pogoreutz, C. , Voolstra, C. R. , Wiedenmann, J. , & Wild, C. (2015). Nitrogen cycling in corals: The key to understanding holobiont functioning? Trends in Microbiology, 23, 490–497. 10.1016/j.tim.2015.03.008 25868684

[mec15298-bib-0049] Rädecker, N. , Raina, J.‐B. , Pernice, M. , Perna, G. , Guagliardo, P. , Kilburn, M. R. , … Voolstra, C. R. (2018). Using Aiptasia as a model to study metabolic interactions in cnidarian‐*Symbiodinium* symbioses. Frontiers in Physiology, 9, 214–214. 10.3389/fphys.2018.00214 29615919 PMC5864895

[mec15298-bib-0050] Rappsilber, J. , Mann, M. , & Ishihama, Y. (2007). Protocol for micro‐purification, enrichment, pre‐fractionation and storage of peptides for proteomics using StageTips. Nature Protocols, 2, 1896 10.1038/nprot.2007.261 17703201

[mec15298-bib-0051] Ray, H. , Suau, F. , Vincent, A. , & Dalla Venezia, N. (2009). Cell cycle regulation of the BRCA1/acetyl‐CoA‐carboxylase complex. Biochemical and Biophysical Research Communications, 378, 615–619. 10.1016/j.bbrc.2008.11.090 19061860

[mec15298-bib-0052] Reaka‐Kudla, M. L. , Wilson, D. E. , & Wilson, E. O. (1997). Biodiversity II: Understanding and protecting our biological resources. Journal of Insect Conservation, 1, 247–250.

[mec15298-bib-0053] Richter, K. , Schmutz, I. , Darna, M. , Zander, J. F. , Chavan, R. , Albrecht, U. , & Ahnert‐Hilger, G. (2018). VGLUT1 binding to endophilin or intersectin1 and dynamin phosphorylation in a diurnal context. Neuroscience, 371, 29–37. 10.1016/j.neuroscience.2017.11.034 29199069

[mec15298-bib-0054] Robles, M. S. , Humphrey, S. J. , & Mann, M. (2017). Phosphorylation is a central mechanism for circadian control of metabolism and physiology. Cell Metabolism, 25, 118–127. 10.1016/j.cmet.2016.10.004 27818261

[mec15298-bib-0055] Rohwer, F. , Seguritan, V. , Azam, F. , & Knowlton, N. (2002). Diversity and distribution of coral‐associated bacteria. Marine Ecology Progress Series, 243, 1–10. 10.3354/meps243001

[mec15298-bib-0056] Rosic, N. , Ling, E. Y. S. , Chan, C.‐K.‐K. , Lee, H. C. , Kaniewska, P. , Edwards, D. , … Hoegh‐Guldberg, O. (2014). Unfolding the secrets of coral–algal symbiosis. The ISME Journal, 9, 844. 10.1038/ismej.2014.182 PMC4817714 25343511

[mec15298-bib-0057] Schmutz, C. , Ahrne, E. , Kasper, C. A. , Tschon, T. , Sorg, I. , Dreier, R. F. , … Arrieumerlou, C. (2013). Systems‐level overview of host protein phosphorylation during *Shigella flexneri* infection revealed by phosphoproteomics. Molecular & Cellular Proteomics: MCP, 12, 2952–2968. 23828894 10.1074/mcp.M113.029918 PMC3790303

[mec15298-bib-0058] Schnitzler, C. E. , & Weis, V. M. (2010). Coral larvae exhibit few measurable transcriptional changes during the onset of coral‐dinoflagellate endosymbiosis. Marine Genomics, 3, 107–116. 10.1016/j.margen.2010.08.002 21798204

[mec15298-bib-0059] Simona, F. , Zhang, H. , & Voolstra, C. R. (2018). Isolation of phosphoproteins from symbiotic and aposymbiotic Aiptasia anemones for elucidation of the phosphoproteome. Protocols.io. 10.17504/protocols.io.txuepnw

[mec15298-bib-0060] Sproles, A. E. , Oakley, C. A. , Matthews, J. L. , Peng, L. , Owen, J. G. , Grossman, A. R. , … Davy, S. K. (2019). Proteomics quantifies protein expression changes in a model cnidarian colonised by a thermally tolerant but suboptimal symbiont. The ISME Journal, 13(9), 2334–2345. 31118473 10.1038/s41396-019-0437-5 PMC6775970

[mec15298-bib-0061] Tyanova, S. , Temu, T. , Sinitcyn, P. , Carlson, A. , Hein, M. Y. , Geiger, T. , … Cox, J. (2016). The Perseus computational platform for comprehensive analysis of (prote)omics data. Nature Methods, 13, 731 10.1038/nmeth.3901 27348712

[mec15298-bib-0062] Venn, A. A. , Tambutté, E. , Lotto, S. , Zoccola, D. , Allemand, D. , & Tambutté, S. (2009). Imaging intracellular pH in a reef coral and symbiotic anemone. Proceedings of the National Academy of Sciences of the United States of America, 106, 16574–16579. 10.1073/pnas.0902894106 19720994 PMC2757848

[mec15298-bib-0063] Voolstra, C. R. (2013). A journey into the wild of the cnidarian model system Aiptasia and its symbionts. Molecular Ecology, 22, 4366–4368. 24137737 10.1111/mec.12464

[mec15298-bib-0064] Voolstra, C. R. , Schwarz, J. A. , Schnetzer, J. , Sunagawa, S. , Desalvo, M. K. , Szmant, A. M. , … Medina, M. (2009). The host transcriptome remains unaltered during the establishment of coral–algal symbioses. Molecular Ecology, 18, 1823–1833. 10.1111/j.1365-294X.2009.04167.x 19317843

[mec15298-bib-0065] Wang, D. , & Dong, X. (2011). A highway for war and peace: The secretory pathway in plant‐microbe interactions. Molecular Plant, 4, 581–587. 10.1093/mp/ssr053 21742620 PMC3146739

[mec15298-bib-0066] Wang, D. , Griffitts, J. , Starker, C. , Fedorova, E. , Limpens, E. , Ivanov, S. , … Long, S. (2010). A nodule‐specific protein secretory pathway required for nitrogen‐fixing symbiosis. Science (New York, NY), 327, 1126–1129. 10.1126/science.1184096 PMC4824053 20185723

[mec15298-bib-0067] Wang, J. , & Douglas, A. (1998). Nitrogen recycling or nitrogen conservation in an alga‐invertebrate symbiosis? The Journal of Experimental Biology, 201, 2445–2453. 9679106 10.1242/jeb.201.16.2445

[mec15298-bib-0068] Wang, Q. , Jiao, F. , Zhang, P. , Yan, J. , Zhang, Z. , He, F. , … Cai, H. et al. (2018). CDK5‐mediated phosphorylation‐dependent ubiquitination and degradation of E3 ubiquitin ligases GP78 accelerates neuronal death in Parkinson's disease. Molecular Neurobiology, 55, 3709–3717. 28528366 10.1007/s12035-017-0579-2

[mec15298-bib-0069] Wiśniewski, J. R. , Zougman, A. , Nagaraj, N. , & Mann, M. (2009). Universal sample preparation method for proteome analysis. Nature Methods, 6, 359 10.1038/nmeth.1322 19377485

[mec15298-bib-0070] Wolfowicz, I. , Baumgarten, S. , Voss, P. A. , Hambleton, E. A. , Voolstra, C. R. , Hatta, M. , & Guse, A. (2016). Aiptasia sp. larvae as a model to reveal mechanisms of symbiont selection in cnidarians. Scientific Reports, 6, 32366. 27582179 10.1038/srep32366 PMC5007887

[mec15298-bib-0071] Wu, R. , Dephoure, N. , Haas, W. , Huttlin, E. L. , Zhai, B. , Sowa, M. E. , & Gygi, S. P. (2011). Correct interpretation of comprehensive phosphorylation dynamics requires normalization by protein expression changes. Molecular & Cellular Proteomics: MCP, 10, M111.009654‐M009111.009654. 10.1074/mcp.M111.009654 PMC3149096 21551504

[mec15298-bib-0072] Xiang, T. , Hambleton, E. A. , DeNofrio, J. C. , Pringle, J. R. , & Grossman, A. R. (2013). Isolation of clonal axenic strains of the symbiotic dinoflagellate *Symbiodinium* and their growth and host specificity. Journal of Phycology, 49, 447–458. 27007034 10.1111/jpy.12055

[mec15298-bib-0073] Xiao, G. H. , Shoarinejad, F. , Jin, F. , Golemis, E. A. , & Yeung, R. S. (1997). The tuberous sclerosis 2 gene product, tuberin, functions as a Rab5 GTPase activating protein (GAP) in modulating endocytosis. The Journal of Biological Chemistry, 272, 6097–6100. 10.1074/jbc.272.10.6097 9045618

[mec15298-bib-0074] Xu, A. , Suh, P. G. , Marmy‐Conus, N. , Pearson, R. B. , Seok, O. Y. , Cocco, L. , & Gilmour, R. S. (2001). Phosphorylation of nuclear phospholipase C beta1 by extracellular signal‐regulated kinase mediates the mitogenic action of insulin‐like growth factor I. Molecular and Cellular Biology, 21, 2981–2990. 11287604 10.1128/MCB.21.9.2981-2990.2001 PMC86927

