# Supplementary material for: Evidence for a role of protein phosphorylation in the maintenance of the cnidarian–algal symbiosis
Source: Mol Ecol. 2019 Dec 6;28(24):5373–86. doi: 10.1111/mec.15298 (PMC6972648; doi:10.1111/mec.15298)
Supplement: Supplementary file 1 [file MEC-28-5373-s001.docx]

**Evidence for a Role of Protein Phosphorylation in the Maintenance of the Cnidarian-Algal Symbiosis**

F Simona^1,*^, H Zhang^2^, CR Voolstra^1,3,*^

^1^ Red Sea Research Center, Division of Biological and Environmental Science and Engineering (BESE), King Abdullah University of Science and Technology (KAUST), Thuwal 23955, Saudi Arabia

^2^ Core Labs, King Abdullah University of Science and Technology (KAUST), Thuwal 23955, Saudi Arabia

^3^ Department of Biology, University of Konstanz, Konstanz 78457, Germany

* co-corresponding authors

**Supplementary Materials and Methods**

### **Total Proteome Sample Preparation**

For total proteome quantification, we processed 20 μg of the total protein homogenate extracted from the aposymbiotic and symbiotic Aiptasia polyps used for phosphoproteomic analysis (5 biological replicates per condition, constituted by pools of 10 small-sized anemones). Protein digestion was performed by Filter-Aided Sample Preparation (FASP). Briefly, the protein extract was loaded in the filter unit, reduced in 10 mM of dithiothreitol (DTT), alkylated in 50 mM of iodoacetamide (IAA), and digested with a Trypsin/Lys-C mix (Cat. V5071; Promega Corp.) at a 1:25 enzyme:protein ratio (w/w), at 37°C overnight. The peptide filtrate was then acidified with TFA (2% by volume) for desalting on a reversed-phase C18 Sep-Pak cartridge (Cat. WAT023590; Waters Corp.), containing an oligo R3 reversed-phase resin (Cat. 1133903; Applied Biosystems), both pre-conditioned in 100% methanol. Peptides were washed twice in 0.1% TFA, eluted in 75% ACN, 0.1% TFA, and completely dried by SpeedVac. The lyophilized peptides were re-dissolved with 0.1% FA in HPLC-grade H_2_0 and quantified by NanoDrop at A_280_. Concentrations were normalized across samples for DIA/SWATH-MS analysis. Indexed retention time (iRT) standards (Cat. Ki-3002; Biognosys) were added to the ready to inject peptide mixture at a 3:10 ratio (v/w).

### **LC-MS/MS Analysis in DIA/SWATH-MS**

Prior to DIA/SWATH-MS, peptides were separated on a nLC system connected to a 50 cm EASY-Spray column PepMap RSLC C18 (Cat. ES803; Thermo Fisher Scientific) at flow rate of 300 nl/min, and over the following 115-min gradient in buffer B (95% ACN): from 2% to 31.5% buffer B in 108 min, ramp up to 95% buffer B in 2 min, maintain 95% buffer B for 5 min, ramp down to 2% buffer B. The nLC system was coupled to a Fusion Lumos Orbitrap mass spectrometer (Thermo Fisher Scientific). The sample was introduced into the MS through an EASY-Spray ion source with an electrospray potential of 1.9 kV, default charge state of 3, ion transfer tube temperature at 275 °C, and activated internal calibration EASY-IC. Full MS scan (375-1400 m/z range) was acquired at a resolution of 120,000 in profile mode and target value of 4xe^5^. The dd-MS^2^ scan was acquired in DIA mode with optimized quadrupole settings for 41 precursor ion selection windows (each 25 Da wide) over the precursor mass range. The isolation window was set at 1.2 m/z, fragmentation by HCD at 30%, orbitrap resolution at 30,000, maximum injection time of 100 ms, and target value of 5xe^4^.

### **DIA Data Processing**

DIA raw data files were imported into Spectronaut X and analyzed with the new spectrum-centric spectral library-free feature of the software (directDIA). Briefly, the software performed a spectrum-centric analysis of the DIA data. A collection of pseudo-MS2 spectra was generated from DIA data by extracting all possible precursor and fragment ion features based on the correlation of the elution profiles. Once the “DIA spectral library” was generated, we ran the quantitative DIA analysis with default settings. Extraction and scoring of MS1 and MS2 mass tolerance as well as retention time (RT) for extracted ion chromatogram (XIC) were set to dynamic and correction factors of 1 were applied. Calibration of iRT standards was done automatically by local (non-linear) regression. Mutated decoys (1 to 3 random amino acids swap) were generated at a library size fraction of 0.1, Q value cutoff (FDR) for precursor and protein identification was set at 0.01, and single protein hits were defined by stripped peptide sequences. Interference correction was applied so that the least interfering 3 fragment ions and 2 precursor ions were kept. All fragment ions not removed during interference correction were used for quantification, calculated from the area under the curve between the XIC boundaries of each targeted ion. Based on the assumption that the majority of the peptides were not regulated (stable background), cross run normalization was performed by Q value sparse, thus all precursors that were identified at least once were used for normalization. Protein group (PG) quantities were inferred from proteotypic peptides and identified with the Aiptasia FASTA database v1.0 (Baumgarten et al., 2015) containing 29,269 entries. We generated a data matrix containing PGs quantified across all samples (5 aposymbiotic, 5 symbiotic) for downstream analysis. Data are available via ProteomeXchange with identifier PXD014076.

## Supplementary Figures


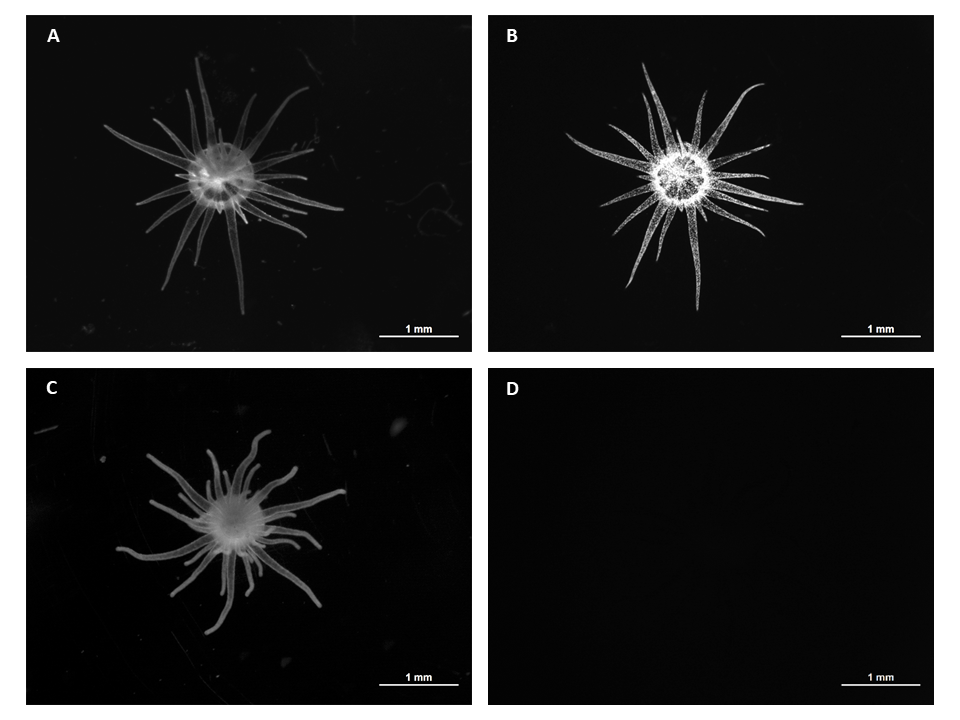


**Figure S1.** Microscopy images of Aiptasia. Symbiotic (A, B) and aposymbiotic (C, D) polyps were imaged under brightfield (A, C) and fluorescent light at 700 nm (B, D) to assess presence or absence of algal symbionts (family Symbiodiniaceae) in anemone host tissue. Visual assessment by fluorescent microscopy was conducted for all 140 anemones used in the experiment, to either confirm the aposymbiotic state or to ensure a comparable rate of infection in symbiotic anemones. A Leica microscope DMI3000 B with a 5x objective and a DFC345 FX camera was used.

**Table S1.** RT-qPCR using clade-specific ITS2 primers

|  | | |  |  |
| --- | --- | --- | --- | --- |
|  | **Clade A primers** | | **Clade B primers** | |
| **Sample name** | **C_T_** | **T_m_ Value** | **C_T_** | **T_m_ Value** |
| A culture | 19.8 | 83.0 | 31.4 | 81.5 |
| A culture | 19.7 | 83.3 | 31.6 | 81.5 |
| B culture | 33.4 | 83.3 | 18.4 | 81.8 |
| B culture | NaN | 83.0 | 18.0 | 81.8 |
| Aposymbiotic Aiptasia | 34.0 | 83.3 | 31.7 | 81.8 |
| Aposymbiotic Aiptasia | NaN | 65.4 | 32.3 | 81.8 |
| SSB01-Aiptasia 1 | NaN | 67.8 | 22.3 | 82.0 |
| SSB01-Aiptasia 1 | NaN | 74.6 | 22.1 | 82.0 |
| SSB01-Aiptasia 2 | NaN | 76.7 | 22.7 | 82.2 |
| SSB01-Aiptasia 2 | NaN | 62.5 | 22.7 | 82.2 |
| SSB01-Aiptasia 3 | NaN | 69.9 | 21.5 | 82.2 |
| SSB01-Aiptasia 3 | NaN | 62.0 | 21.0 | 82.2 |
| No DNA template | NaN | 62.3 | 32.3 | 82.2 |

Sequences (5’-3’) of the primers used (Correa et al., 2009): Clade A forward 5’-CCTCTTGGACCTTCCACAAC-3’, reverse 5’-GCATGCAGCAACACTGCTC-3’; Clade B forward 5’-GTCTTTGTGAGCCTTGAGC-3’, reverse 5’-GCACACTAACAAGTGTACCATG-3’. qPCR program: 50°C for 5 minutes hold, 95°C for 2 minutes hold, 40 cycles of 95°C for 3 seconds and 60°C for 30 seconds. C_T_: cycle threshold, T_m_: melting temperature. For the experimental animals, validation by qPCR was not performed since the amount of sample was limited: generation of the phosphoproteome required a high amount of total protein extract (750 ug – 1.5 mg).


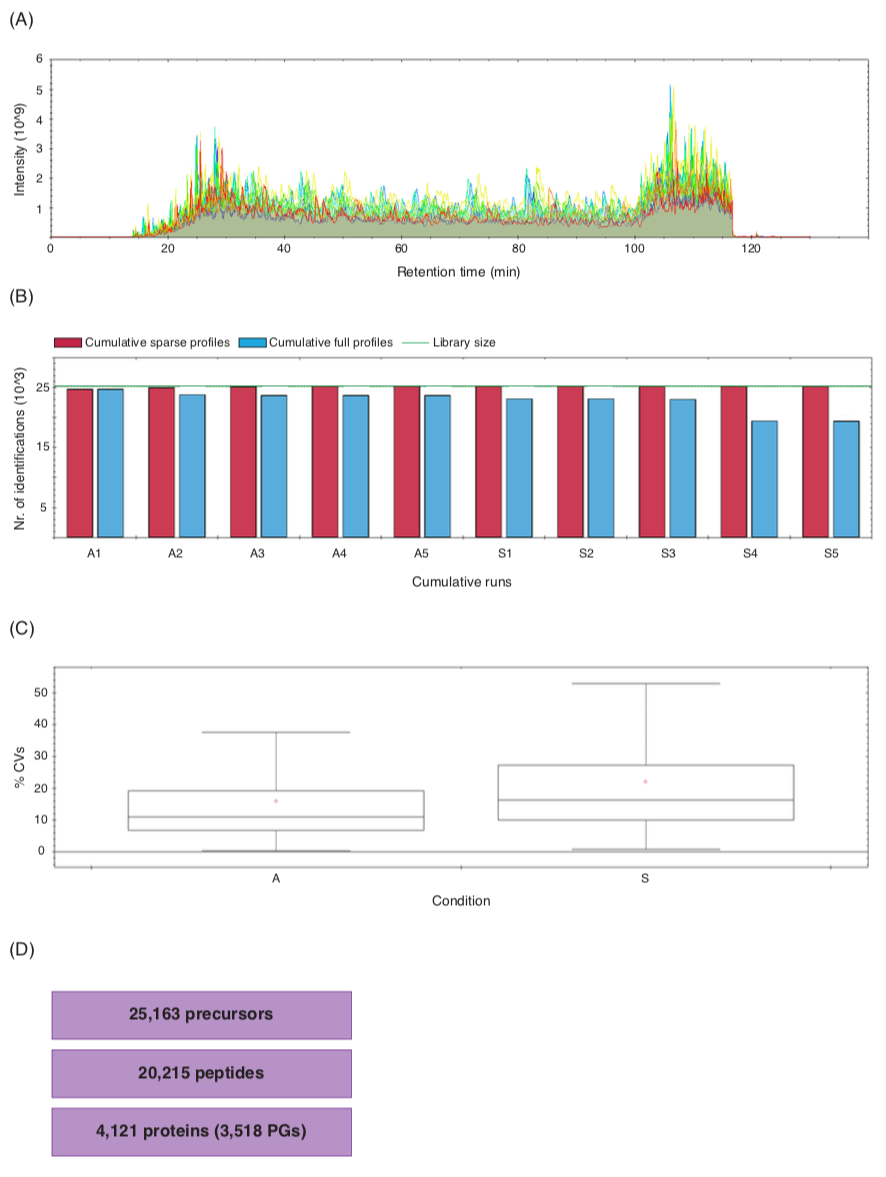


**Figure S2.** Total proteome DIA/SWATH-MS analysis. (A) total ion chromatogram (TIC) of all samples shows reproducibility of the MS runs. (B) Total proteome quantification by directDIA. A DIA spectral library was first generated (green line) from the cumulative sparse profiles (red bars), namely from all the peptides that were identified at least once across all samples. The cumulative full profiles (blue bars) represented the peptides that were consistently detected across all samples. (C) The boxplot represents the coefficient of variation (CV) within symbiotic states. (D) Number of precursors, peptides, and proteins (clustering in PGs) that were considered for total proteome quantification. Peptide-spectrum match (PSM), peptide, and protein FDRs set to 0.01.


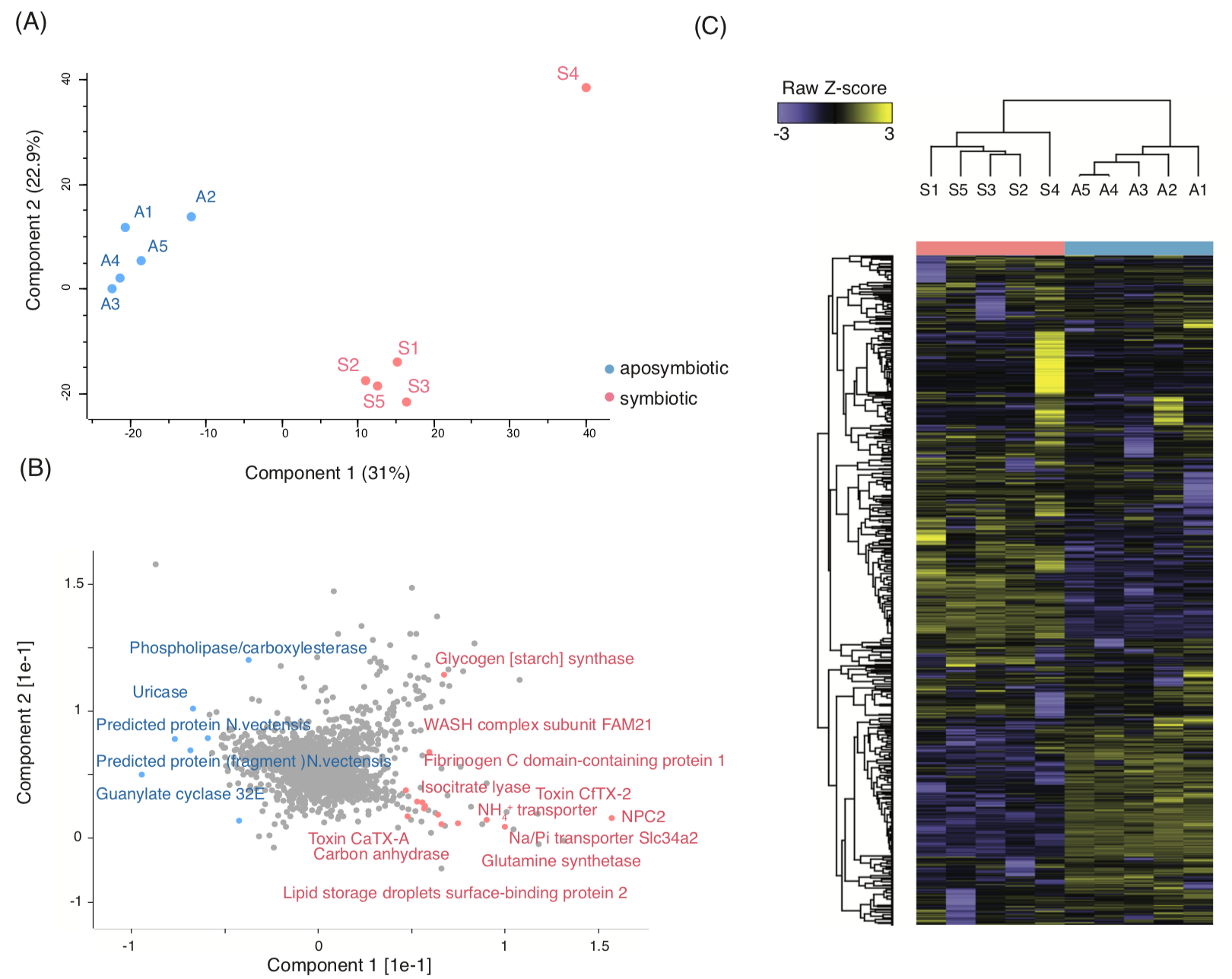


**Figure S3.** Proteomic profiling of aposymbiotic (A, blue) and symbiotic (S, pink) samples. Principal component analysis (PCA) representing (A) the major components determining distance across samples and (B) the proteins defining these components. The names of some candidate proteins commonly described in cnidarian-algal symbioses are highlighted. (C) Unsupervised Pierson’s hierarchical clustering showing samples profiles.

**
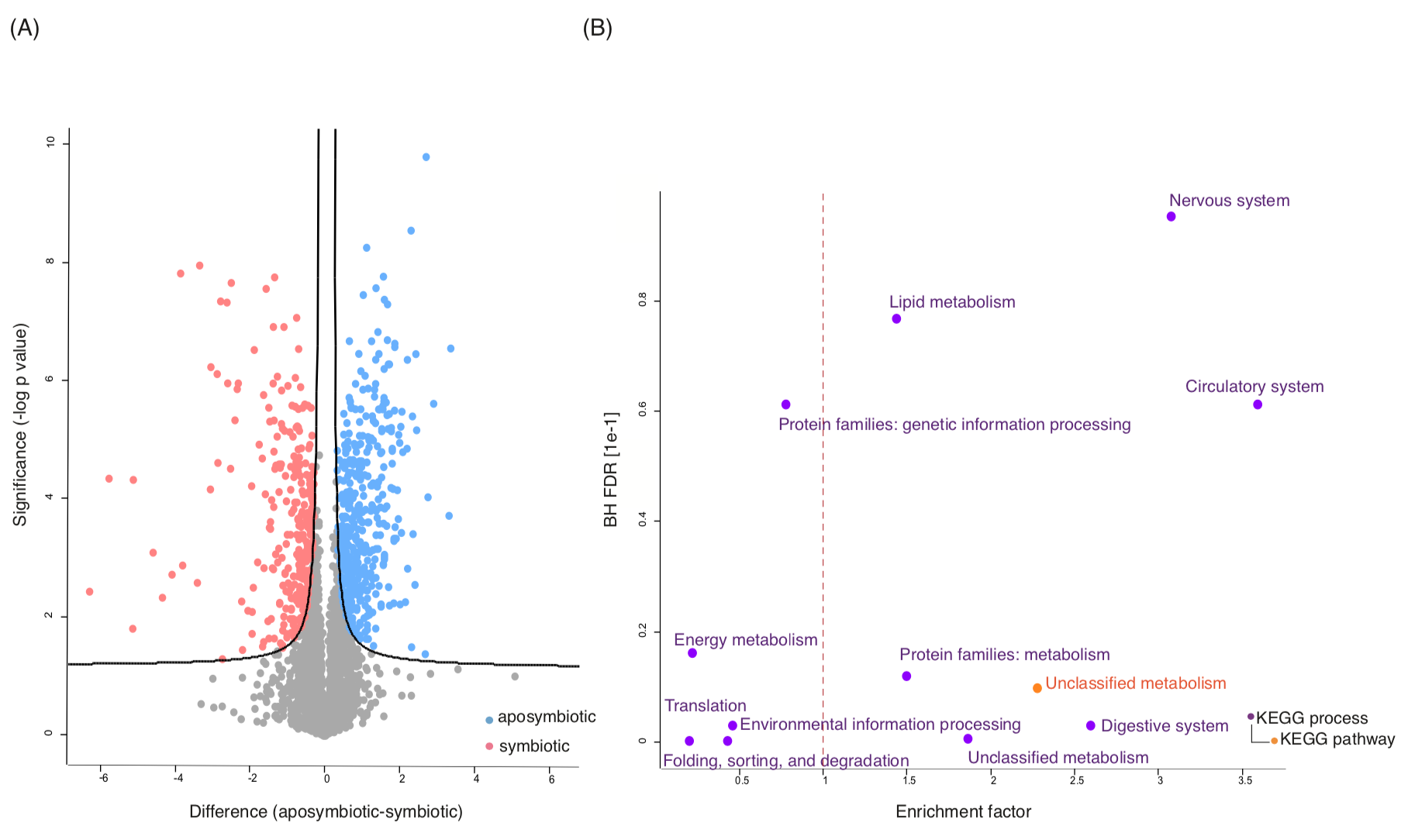
**

**Figure S4.** Differentially abundant proteins (737 PGs) and enrichment analysis of KEGG processes and pathways targeted by differential protein expression. (A) Volcano plot representing highly abundant proteins in symbiotic (pink, left side) or aposymbiotic (light blue, right side) Aiptasia anemones (FDR 0.01, S0 0.1). (B) Scatterplot representing significantly enriched KEGG terms in the total proteome (FDR < 0.01). KEGG terms are hierarchically classified into processes (purple) and pathways (orange). Underrepresented KEGG terms are found at the left of the red dashed line with an enrichment factor <1.

**
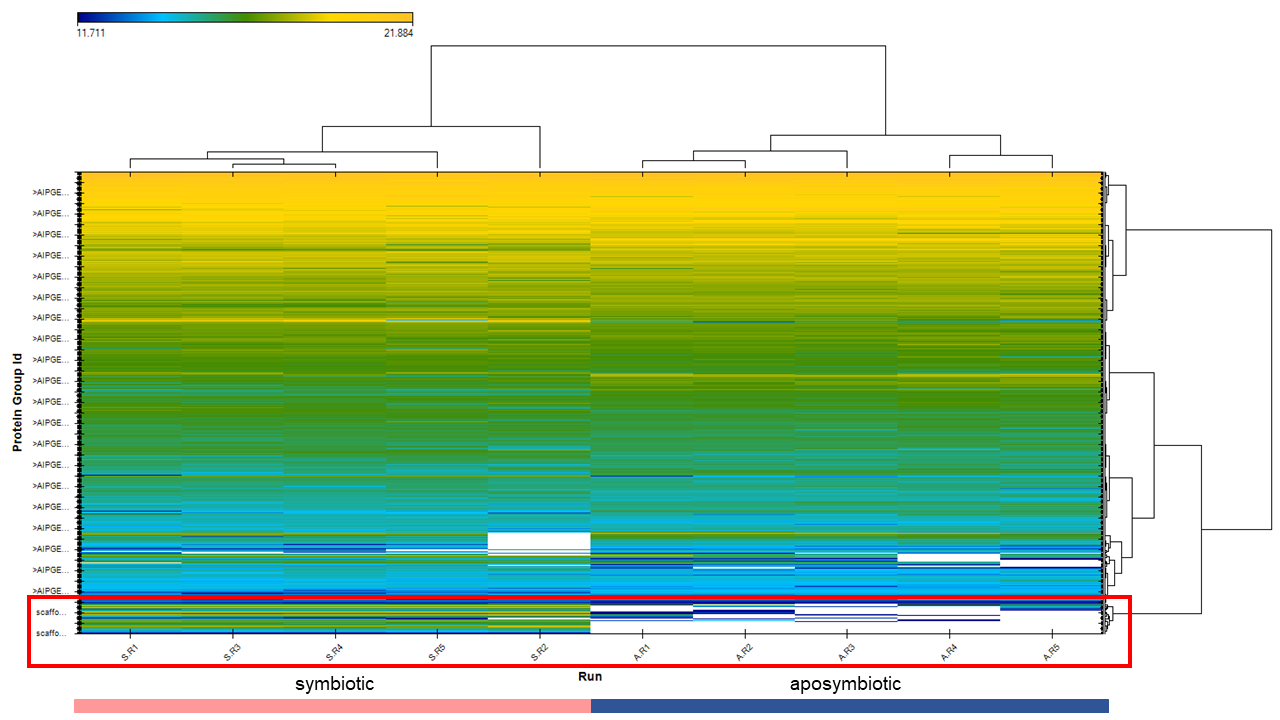
**

**Figure S5.** Proteomic profiling of symbiotic (pink) and aposymbiotic (blue) Aiptasia samples using host and symbiont protein databases: host (protein id: AIPGENES) and symbiont (protein id: scaffolds). The host database served as a stable background for calibration and normalization of the data. In the symbiotic samples, the *Breviolum* protein groups clustered together (red rectangle), while in the aposymbiotic samples, no or sparse proteins were detected in the corresponding cluster. The data matrix of the heatmap is available as Supplementary File S5.

## Supplementary Data

**Dataset S1.** List of quantified and differentially abundant phosphopeptides between aposymbiotic and symbiotic Aiptasia anemones.

**Dataset S2.** List of phosphoproteins included in the KEGG enrichment analysis.

**Dataset S3.** List of quantified and differentially abundant proteins between aposymbiotic and symbiotic Aiptasia anemones.

**Dataset S4.** List of proteins included in the KEGG enrichment analysis.

**Dataset S5.** Data matrix for generation of the Aiptasia-*Breviolum* heatmap.
